# Physical constraints and environmental factors shape phloem anatomical traits in woody angiosperm species

**DOI:** 10.1111/nph.70578

**Published:** 2025-09-15

**Authors:** Yan Wang, Johannes Liesche, Alan Crivellaro, Jiří Doležal, Jan Altman, Donato Chiatante, Anastazija Dimitrova, Zexin Fan, Peili Fu, Félix Forest, Jozica Gričar, Patrick Heuret, Sandrine Isnard, Olivier Maurin, Antonio Montagnoli, Cyrille B. K. Rathgeber, Enkhchimeg Tsedensodnom, Santiago Trueba, Yann Salmon

**Affiliations:** ^1^ Institute for Atmospheric and Earth System Research/Physics, Faculty of Science University of Helsinki P.O. box 68, Gustaf Hällströmin katu 2b Helsinki 00014 Finland; ^2^ Institute for Atmospheric and Earth System Research/Forest Sciences, Faculty of Agriculture and Forestry University of Helsinki P.O. Box 27, Latokartanonkaari 7 Helsinki 00014 Finland; ^3^ Institute of Biology University of Graz Graz 8010 Austria; ^4^ Department of Agricultural, Forest and Food Sciences University of Torino Largo Paolo Braccini 2 Grugliasco (TO) 10095 Italy; ^5^ Forest Biometrics Laboratory, Faculty of Forestry ‘Stefan cel Mare’ University of Suceava Str. Universitatii 13 Suceava 720229 Romania; ^6^ Institute of Botany, Czech Academy of Sciences Dukelská 135 Třeboň 37901 Czech Republic; ^7^ Department of Botany, Faculty of Science University of South Bohemia Branišovská 1760 České Budějovice 837005 Czech Republic; ^8^ Faculty of Forestry and Wood Sciences Czech University of Life Sciences Prague, Kamýcká 129 Prague 16521 Czech Republic; ^9^ Laboratory of Environmental and Applied Botany, Department of Biotechnology and Life Science University of Insubria Via Dunant 3 Varese 21100 Italy; ^10^ Hans Em Faculty of Forest Sciences, Landscape Architecture and Environmental Engineering Ss. Cyril and Methodius University in Skopje Skopje 1000 North Macedonia; ^11^ CAS Key Laboratory of Tropical Forest Ecology Xishuangbanna Tropical Botanical Garden, Chinese Academy of Sciences Mengla Yunnan 666303 China; ^12^ Ailaoshan Station of Subtropical Forest Ecosystem Studies Xishuangbanna Tropical Botanical Garden, Chinese Academy of Sciences Jingdong Yunnan 676209 China; ^13^ Royal Botanic Gardens Kew TW9 3AE UK; ^14^ Department of Forest Physiology and Genetics Slovenian Forestry Institute Ljubljana 1000 Slovenia; ^15^ AMAP, University of Montpellier, CIRAD, CNRS, INRAE, IRD Montpellier 34398 France; ^16^ Meise Botanic Garden Meise 1860 Belgium; ^17^ Fédération Wallonie‐Bruxelles Service Général de l'Enseignement supérieur et de la Recherche scientifique Bruxelles 1080 Belgium; ^18^ Université de Lorraine, AgroParisTech, INRAE, SILVA Nancy F‐54000 France; ^19^ Laboratory of Forest Genetics and Ecophysiology, School of Engineering and Applied Sciences National University of Mongolia Ulaanbaatar 14201 Mongolia

**Keywords:** adaptation, allometry, phloem sieve element, tip‐to‐base conduit widening, xylem vessel

## Abstract

- Xylem trait studies have enhanced our understanding of how plants strategically adapt their morphological and anatomical features to diverse climates. Despite the importance of the phloem in plant functioning, similar studies of phloem traits are lacking. To tackle this knowledge gap, we analyzed phloem anatomical traits of woody angiosperm species in relation to climate and the distance of samples to the stem tip.
- We collected main stem or branch cross‐sections of 188 angiosperm woody species, which represent a wide range of climates and diverse families. Measurements of xylem vessel and phloem sieve element diameter, density, and lumen fraction were used in phylogenetic structural equation models to disentangle internal and climatic constraints on their morphological and anatomical features.
- Our results showed that distance‐to‐tip mainly affects sieve element and vessel diameter and density, while climate more strongly influenced conduit lumen fraction. Vessel size was positively correlated with temperature after correcting for the distance‐to‐tip, while sieve element diameter was correlated with water availability.
- Our results highlight the need to account for distance‐to‐tip when accessing anatomical variations linked to the environment, and show that sieve element traits respond to other climatic drivers than vessel traits rather than simply mirroring them.

## Introduction

Vascular plants have evolved two systems for long‐distance sap transport: xylem, which transports water and nutrients upwards from the roots to the canopy leaves, and phloem, which transports photosynthesis‐derived carbohydrates from leaves and other source organs to sink organs, such as cambium, flowers, and roots (Evert, 1982). These two transport systems are essential for maintaining the water and carbon balance in the plant and hence its fitness. Several xylem anatomical features, such as xylem conduit lumen area and density, were shown to be related to the plant's ability to adapt to environmental factors; for example, by contributing to its ability to survive drought (Qaderi *et al*., 2019) and other environmental stressors, such as freezing (Lintunen *et al*., 2018). Despite its importance in photosynthate distributions throughout the plant, the role of phloem structure in defining plant ecological functions has only been superficially investigated (Tang *et al*., 2022). This may stem from the fragility and strong wound reaction of the tissue, making it susceptible to damage and, thus, difficult to experimentally study its function or analyze its structure using imaging (Sevanto, 2019). Given that phloem transport has been suggested as a key process for understanding and predicting plant susceptibility and responses to drought (Savage *et al*., 2016; Sevanto, 2018) and cold stress (Peuke *et al*., 2006; De Schepper *et al*., 2011), it is crucial to investigate phloem structure across plant taxa and environments.

Theoretical work suggests that the phloem transport system needs to couple with the xylem transport system as water is expected to flow from one to the other to equilibrate water potential in both tissues (Hölttä *et al*., 2009). However, experimental data are still lacking to test whether phloem properties and dimensions are coordinated to match the xylem conduction properties or whether they are differentially scaled and influence species performance in contrasting environments. Xylem traits have been studied to reveal plant strategies to cope with changing environments (Castagneri *et al*., 2017) and linked to species distribution across different climates (Borghetti *et al*., 2020; Maharjan *et al*., 2021). Phloem traits are more difficult to measure and, as a result, have barely been investigated in relation to plant presence and climate. Therefore, whether they can provide additional information about plant strategies to adapt to their environment remains an open question.

Furthermore, if phloem traits closely mirror xylem traits, it may be possible to infer them from existing xylem trait datasets, offering a less labor‐intensive alternative. Otherwise, direct measurement of both tissues would be required despite the greater effort involved. It is well established that tree height or sample cross‐section surface area needs to be considered as controlling factors when studying conduit size‐related traits, since the conduits widen farther from the stem tip (Anfodillo *et al*., 2013; Olson *et al*., 2021). As a result, the xylem conduit diameter is primarily explained by the hydraulic path length, rather than the growing environment of the plant (Olson *et al*., 2018). Xylem conduit widening is an adaptive response to minimize the hydraulic resistance along the path, as xylem conduit resistance can be calculated by adding the resistance of each segment of the conduits, which is proportional to the conduit length and the reciprocal of the fourth power of conduit radius (Olson *et al*., 2021). Conduits at the bottom of the stem must be sufficiently wide to compensate for the resistance along the full hydraulic path (Koçillari *et al*., 2021). Olson *et al*. (2021) proposed an exponential relationship between xylem conduit size and the distance‐to‐tip. This model can predict within‐species conduit diameters in both angiosperm and gymnosperm species (Petit & Crivellaro, 2014; Williams *et al*., 2019).

Sieve elements, the main phloem cells involved in distributing photosynthesis products, are elongated and axially connected via sieve plates in angiosperms. In gymnosperm species, phloem sieve cells do not possess sieve plates and are connected via sieve areas. They lack rigid walls and contain living protoplasts under positive hydrostatic pressure (Evert, 1982). Phloem sieve element sizes may be tied to xylem conduit sizes across different species (Tang *et al*., 2022) for the following reasons: (1) growth conditions: sieve elements in the phloem should widen to align with the xylem conduits, as expansion of cells is closely related to turgor pressure during secondary growth (Ali *et al*., 2023); and (2) coordination of resistance in transport systems: phloem and xylem transport are coupled according to the Münch hypothesis (Hölttä *et al*., 2006). Efficient xylem water transport should support higher photosynthesis (Liu *et al*., 2019), which in turn would require efficient phloem transport (Dewar *et al*., 2022). Such coordination of phloem transport and photosynthetic capacity was shown in leaves of nonwoody species (Adams *et al*., 2013, 2022). Sieve element resistance is considered mainly from plate resistance and lumen resistance, and although other potential sources of resistance, such as protein, might be present (Hao *et al*., 2008; Knoblauch *et al*., 2014), they have remained mainly unaccounted for in morphological studies. Lumen resistances are typically lower than sieve element plate resistance, but both lumen and plate resistance were shown to be correlated (Jensen *et al*., 2012). Furthermore, the total resistance of the conduit is expected to be positively correlated to the length of the conduit when the full plant is considered, as plate number increases with total path lengths. The lumen resistance is also inversely proportional to sieve element radius and accumulates along the path (Savage *et al*., 2017). Consequently, lumen resistance can be used as a proxy for total phloem transport resistance, and plants must also produce wider phloem conduits at the bottom to compensate for accumulated resistance and enable long‐distance transport (Knoblauch *et al*., 2016). Sieve element widening in stems of woody plants has been investigated in relatively few species (Petit & Crivellaro, 2014; Savage *et al*., 2017; Kiorapostolou & Petit, 2019; Clerx *et al*., 2020). Studies based on diverse phylogenetic groups, especially under the frame of climate–trait interaction, are thus needed to understand how environmental factors influence phloem anatomy across lineages. Such studies would provide insights into the adaptive significance of sieve element dimensions in relation to hydraulic architecture and climate, contributing to a more comprehensive understanding of plant transport strategies and their evolutionary drivers.

Although the main driver of xylem conduit diameter by far is hydraulic path length (Olson *et al*., 2021), certain climate factors still have a non‐negligible influence on xylem vessel diameters alongside distance to the stem tip across angiosperm species. Recent studies indicated that, after accounting for distance to the tip, annual temperature, potential evaporation, and vapor pressure deficit were associated with xylem vessel diameters, with wider vessels occurring in warmer environments and lower evaporative demand (Olson *et al*., 2014, 2021). Interestingly, water availability does not show constant influence on xylem vessel size: When tree height was considered, the relationship between precipitation and vessel size either changed within species (Fajardo *et al*., 2020) or disappeared across angiosperms (Olson *et al*., 2014). However, another study argued that Eucalypt species still produced small vessels at sites with lower water availability (Pfautsch *et al*., 2016). Wide xylem conduits are more efficient for water transport, but they also tend to be more vulnerable to embolism. This is consistent with the observed positive correlation between xylem vessel width and mean annual precipitation within species (García‐Cervigón *et al*., 2018) and across species (Warwick *et al*., 2017). However, as stated earlier, xylem vessel diameter is strongly related to the distance‐to‐tip, suggesting that the relationships found without considering the distance‐to‐tip could be confounded by this factor.

Whether phloem anatomical traits respond to climate factors across species similarly to xylem traits remains to be investigated. Indeed, phloem and xylem's transport cells at functional maturity strongly differ: Xylem vessels are dead cells, whereas sieve elements are living cells. As a result, transport in the phloem occurs in the symplast, unlike transport in the xylem. They also differ in the mechanisms driving transport: water transport in xylem is driven by the transpiration in the leaf, which allows water to overcome gravity and friction as the water column is kept together by the cohesion force between water molecules (Konrad *et al*., 2019). This friction arises from both interactions between water molecules and conduits, as well as between water molecules themselves (Konrad *et al*., 2019). In Münch theory (Münch, 1930, as cited in Knoblauch *et al*., 2016), phloem transport results from the osmotic difference between source and sink organs. Sugars are loaded from the mesophyll in the phloem. The loading of sugars increases osmotic pressure in the phloem cells in the leaves. As a result, water is driven from the xylem to the phloem in the leaves. Conversely, sugar unloading near carbon sinks has the opposite effect, resulting in a difference in hydrostatic pressure that leads to phloem sap movement from carbon sources to sinks. Therefore, phloem and xylem transport cells' response to climate could be expected to differ. For instance, in xylem vessels with secondary cell walls, transport failure can occur abruptly as a direct mechanical response. By contrast, phloem sieve element transport declines more gradually by big protein deposition at the sieve plate, for example callose (Mullendore *et al*., 2010), forisomes in legume species (Paulmann *et al*., 2021) and other nonforisome P‐proteins (Noll *et al*., 2022). The extent to which individual sieve elements become occluded and what fraction of sieve elements become nonfunctional in response to environmental conditions can only be estimated from bulk flow measurements (Kalmbach & Helariutta, 2019). In addition to the blocking of sieve plates, earlier studies have suggested that, for some species, extreme drought or cold may directly influence phloem transport by increasing sap viscosity (Epron *et al*., 2016; Hesse *et al*., 2019) and consequently decreasing the overall phloem transport rate (Nakad *et al*., 2022). Sevanto (2018) proposed that under drought conditions, plants should have wider sieve elements as an adaptation to compensate for the high viscosity. By contrast, Salmon *et al*. (2019) suggested that low water availability results in narrower sieve elements due to reduced turgor pressure. Wider sieve elements should improve transport efficiency as lumen resistance is lower. However, the potential cost associated with having wider sieve elements remains unknown. A structural limitation probably exists as the capacity for the exchange of proteins with the companion cells becomes limiting when the radius of the sieve element reaches a threshold (Oparka & Turgeon, 1999). Sieve cells in leaves of some pine trees were shown to have a diameter below the optimal size for efficient phloem loading, suggesting that having wide sieve cells is associated with a cost or a constraint (Liesche *et al*., 2021). Despite existing studies on seasonal variation (Prislan *et al*., 2013 ) and intra‐specific variation (Dannoura *et al*., 2019; Gričar *et al*., 2024) of phloem sieve element diameters, an understanding of whether sieve element anatomical traits respond to climate factors similarly to xylem vessel traits across angiosperm species and whether this response involves a trade‐off is still lacking.

Our goal was to test whether phloem anatomy is influenced by climate when considering the distance to the stem tip. To achieve this, we collected stem cross‐section samples and tested which factors primarily influence the size of sieve elements by addressing the following questions:

1. Do phloem sieve element size, density, and lumen fraction, as well as those of xylem vessels, scale with the distance to the tip across angiosperm species?
2. After controlling for the distance to the tip, do sieve element traits respond to environmental factors?
3. If so, do sieve element traits coordinate with xylem vessel traits in relation to the environment?

We focused exclusively on self‐supporting woody angiosperm species so as not to confound our observations with anatomical downregulation imposed by alternative selection pressures, such as changes in biomechanical status in lianas or responses of nonwoody anatomies.

## Materials and Methods

### Plant material collection and species selection

We obtained existing anatomical cuttings processed in different laboratories from fresh materials. Cross‐section images were available for 188 species, covering 154 genera, 64 families, and 25 orders from tropical, subtropical, Mediterranean, and temperate climates in Europe, Asia, South America, and Africa (Supporting Information Table S1). Forty‐three species had two to five replicates, while the remaining species had only one sample each. We obtained cutting slides of 176 species and digitalized images using a light microscope. Images of the remaining species had already been obtained before this study. We obtained main stem samples from 157 species and branch samples from 75 species, with 44 species sampled for both. For each individual, only one sample per organ type was obtained. When samples were taken from the main stem, the distance‐to‐tip was calculated as tree height minus the sampling distance from the base. When samples were taken from the branch, the distance‐to‐tip was measured from the branch tip to the sampling position. In cases where both stem and branch samples were taken from the same individual, only one height was used for each type of organ sample. We selected 303 samples from which both xylem and phloem were intact. Anatomy cuttings were prepared in different laboratories, and the methods are shown in Table S2. Our sampling spanned a range of plant height from 0.13 to 45 m. The sampling locations covered a wide range of climatic conditions: annual mean temperature varied from 1.75°C to 26.35°C, and annual precipitation ranged from 212 to 6129 mm. Samples were taken from 0 to 2204 m above sea level.

### Xylem and phloem conduit trait measurements

**Fig. 1:**
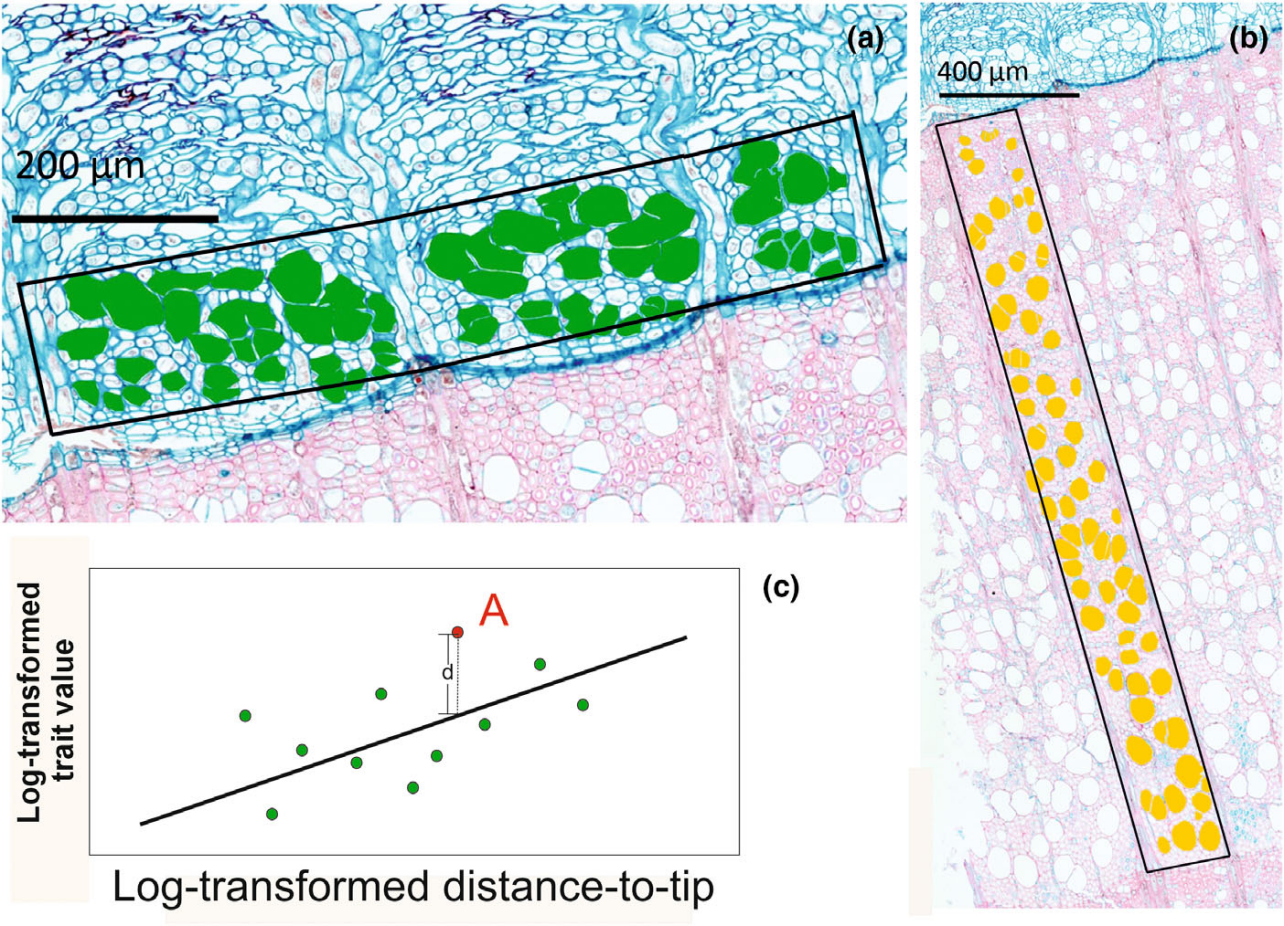
Anatomical feature. (a) Sieve element lumen area (in green), density, and fraction measurement. (b) Xylem vessel lumen area (in yellow), density, and fraction, using *Fagus sylvatica* L. as an example. (c) Detrended value. The black solid line represents linear regression between log‐transformed trait and log‐transformed distance‐to‐tip. For a given species A (red dot in the figure), the detrended trait value is the residual (dashed line d), which means the distance between raw value and the predicted value.

We manually outlined xylem vessels and phloem sieve elements using the ROI Manager in imagej (v.1.54g, Schneider *et al*., 2012), and their lumen areas were then measured within the software (Fig. 1a,b). For each species, we obtained one or two images for each cross‐section. For ring‐porous species and diffuse‐porous species with clear ring boundaries, we selected a xylem annular sector between at least two rays in the outermost ring layer and measured the lumen area of all the xylem vessels (at least 50) inside of the target area to represent both early‐ and latewood. For 16 species sampled in the early growing season in which the last xylem ring was incomplete (current year at cutting), we measured the xylem vessel traits in the last fully formed ring (year before cutting). We selected a random area from the outer xylem layer for species without clear boundaries. We calculated the equivalent xylem vessel diameter (*D*) using Eqn (1) for each xylem vessel, where *A* is the lumen area.

(Eqn 1) $$D=\sqrt{\frac{4A}{\pi }}$$

We then calculated the median of the xylem vessel diameter and the first and third quartiles of the xylem vessel diameter. Hydraulic diameters (*D*_H_) were calculated using Eqn (2),

(Eqn 2) $$D_{H}={\left(\frac{\sum D^{4}}{N}\right)}^{\frac{1}{4}}$$

where *N* is the number of measured vessels. *D*_*H*_ represents the equivalent mean diameter that vessels in a stem would need to have to maintain the same overall conductivity for a given number of conduits. Both formulas are detailed in Scholz *et al*. (2013). We counted the total vessel number in the target sector and divided it by the area of the sector to calculate vessel density. Xylem vessel lumen fraction was calculated as the ratio of total xylem vessel lumen area in the target area to the total surface of the sector (Fig. 1b). Density and fraction were obtained only from main stem samples.

We measured at least 25 sieve element lumen areas within the conductive phloem of the same cross‐section and calculated the equivalent diameter following Eqn (1). Conductive phloem was identified in an area with noncollapsed sieve elements and before the dilation of rays or parenchyma cells (Angyalossy *et al*., 2016; Fig. 1a). We identified sieve elements by the following criteria: (1) surrounded by companion cells, (2) the presence of sieve plates, and (3) using the shape or position information for the same species available in published material, for example textbooks, atlas of woody species anatomy (some examples are shown in Fig. S1). We then calculated the sieve element *D*_*H*_, median, upper quartile, and lower quartile diameters. We also selected an area between at least two rays in the conductive phloem to calculate the sieve element density and fraction (Fig. 1a). Sieve element density and fraction were calculated following the protocols previously described for vessel measurements in xylem, and they were obtained only from main stem samples due to the difficulty in distinguishing sieve elements from phloem parenchyma in the branch cross‐sections. We obtained sieve element density and fraction from main stem samples in 84 of the 156 species due to difficulty in distinguishing sieve elements from parenchyma in some species in cross‐sections. For comparable results, we analyzed vessel density and fraction in xylem on the same sample subset.

### Data and statistical analyses

All data analyses were conducted in R (R Core Team, 2024). We conducted separate analyses for branch and main stem samples. We ran a generalized least square model with the function gls() from the package nlme (Pinheiro *et al*., 2025) to analyze the regression of all the traits against distance‐to‐tip. We estimated the scaling exponent (i.e. the slope of the trait–distance relationship) and the intercept for each anatomical trait and tissue. To account for phylogenetic effects, we compared models with (pGLS model) and without (GLS model) phylogeny correction using the ape package to estimate Pagel's λ (Paradis & Schliep, 2019). Pagel's λ is a phylogeny scaling factor that quantifies the extent to which shared evolutionary history explains the similarity patterns observed in data (Pearse *et al*., 2025). Model selection was based on the Bayesian information criterion (BIC). We examined the variance structure alongside phylogenetic covariance, tested different variance covariates, and retained the model with the lowest BIC value. For pGLS models, we calculated partial *R* ^2^ based on reduced models excluding log(distance) as a predictor while maintaining the same variance structure both with and without phylogeny correction (lambda = estimated vs lambda = 0; Ives, 2019). For GLS models, we only calculated *R* ^2^ based on reduced models without accounting for phylogeny. Before conducting all the analysis, we compared whether samples from different laboratories exhibited different regression relationships between traits and distance‐to‐tip (log (Traits)~log(distance)). We initially ran a simple linear model on the regression of all traits from each laboratory separately and compared the results with those from the combined dataset. Since the species numbers per laboratory were unbalanced (Table S1), we restricted this analysis only to the three laboratories that provided more than five species. Except for the slope of xylem vessel median diameter and lower quantile diameter, the 95% confidence intervals (CIs) for both intercept and slope from individual laboratories overlapped with the corresponding parameters from the full model (Table S3). Consequently, we grouped all the samples for the subsequent analyses.

We use pGLS to take into account the expected lack of independence of observations across different species caused by the phylogenetic relatedness. The phylogenetic trees were based on Zuntini *et al*. (2024), accounting for different topological uncertainties in unresolved species (Forest, 2023) and were pruned to meet our needs. We obtained 83 binary trees and ran analyses separately on each tree to minimize the error introduced by potential inaccuracy in phylogenetic topology (Adams *et al*., 2025). As the results were robust, we reported the mean parameters estimated across 83 phylogenetic trees. Species names were validated using the World Checklist of Vascular Plants with R package rwcvp (Brown *et al*., 2023). Hybrids were assigned the same name as one of the parent species for phylogenetic correction (Table S1). Eighty‐three phylogenetic trees were used for our analyses (see Data availability). To test whether vessel and sieve element traits scaled similarly with distance‐to‐tip, we compared 95% CIs of the slopes. For a given trait, if the 95% CIs of the slopes did not overlap between vessels and sieve elements, we considered the trait as having a different scaling exponent.

To test whether vessel and sieve element traits were correlated, we used phylogeny‐corrected Pearson's product–moment correlation to explore the correlation among all traits in the main stem sample (package phytools, Revell, 2012). To calculate the phylogenetic Pearson r, we used the phyl.vcv() function in the R package phytools (Revell, 2012) to obtain the phylogenetic trait variance–covariance matrix. The joint lambda for the variance–covariance matrix was obtained by maximizing the likelihood that was calculated with the likMlambda() function (phytools, Revell, 2012). We performed this analysis on both the raw trait values and the detrended value, obtained as the residuals of previous pGLS or GLS (log (Traits)~log(distance)). If, for a given species, the detrended value of a trait (e.g. sieve element size) is positive, it means that for a given distance‐to‐tip, this species has a larger sieve element size than predicted by the previous pGLS or GLS model (Fig. 1c).

**Fig. 2:**
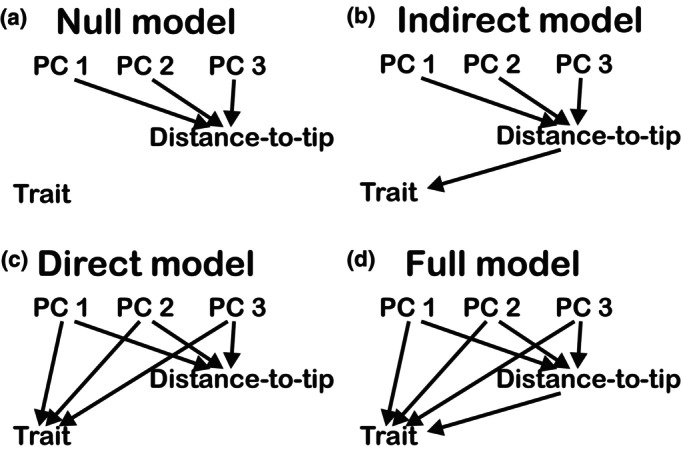
Structural equation model scenario. Arrows represent the influence of climate factors (PCs) or distance‐to‐tip on trait values. (a) A null model supposes that there is no influence of either climate (PCs) or the distance to the tip. (b) An indirect model supposes the influence of the climate is on distance‐to‐tip and then subsequently influences the trait value. (c) A direct model assumes that the climate has impacts on trait values; there is no correlation between the distance‐to‐tip and trait value. (d) A full model allows both direct and indirect influences.

To test whether climatic factors influence traits in addition to the distance‐to‐tip, we extracted 19 bioclimatic variables, which are derived variables from the monthly mean, max, mean temperature, and mean precipitation values, for each main stem sample location from CHELSA (Climatologies at high resolution for the earth's land surface areas) at 30 s‐arc resolution (v.2.1, Brun *et al*., 2022). CHELSA is global climate data based on a mechanistical terrain‐based downscaling of global reanalysis data. We ran a principal component analysis (PCA) to reduce the number of variables and keep the key information (Jolliffe & Cadima, 2016) and chose the first three PCs to represent these 19 climate variables. We first tested how much variance in traits could be explained by the climate alongside distance‐to‐tip by partial regressions. For all the traits, we set the detrended trait value as the response variable and three climate PCs as explanatory variables (detrended value ~ PC1 + PC2 + PC3) and we performed GLS analyses to evaluate the effects of climate on trait variation. Additionally, as an alternative approach, we built four phylogenetic corrected structural equation models (pSEM, package phylopath, van der Bijl, 2018) to evaluate direct and indirect climate influence on each anatomical trait separately. We treated the anatomical trait as the response factor, and we assumed a common path for all models where climate influenced plant height (Fig. 2). We did so because in our study distance‐to‐tip in main stem samples is highly correlated to plant height, a variable known to respond strongly to climate (Seynave *et al*., 2008; Messaoud & Chen, 2011). In the null model, climate did not influence the considered trait (Fig. 2a). The indirect model assumed that the environment impacts distance‐to‐tip (plant height) and then subsequently influenced the trait value; that is the environment does not impact traits directly (Fig. 2b). The direct model assumed that only the environment influenced the trait (Fig. 2c). Finally, we also tested a full model with both direct and indirect influence of the environment on the traits considered (Fig. 2d). We selected the best model for each trait based on C‐statistic information criterion (CICc). Before the analyses, all data were scaled and centered from −1 to 1.

## Results

### Both vessel and sieve element diameter increases with distance‐to‐tip

Xylem vessel median diameters of our samplings in the main stem were from 11.68 to 255.20 μm, and from 9.13 to 78.50 μm in the branch samples. Phloem sieve element median diameters were from 4.63 to 42.19 μm in main stem samples and from 3.78 to 19.77 μm in branch samples.

**Fig. 3:**
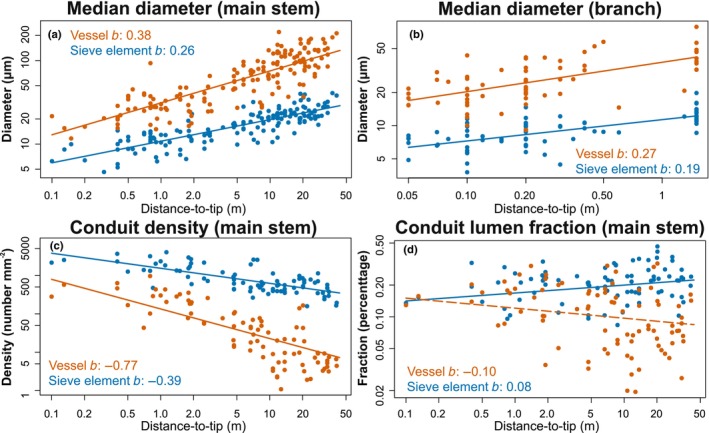
Scaling factors for (a) median diameter in the main stem sample, (b) median diameter in branch sample, (c) vessel and sieve element conduit density, and (d) vessel and sieve element conduit lumen fraction with distance‐to‐tip. The scaling factors b are shown in each graph. Solid line: slope b is significantly different from zero (*P* < 0.05). Dashed line: slope b is not significantly different from zero (*P* ≥ 0.05).

When all the species were pooled together, log‐transformed median xylem vessel diameters increased with the log‐transformed distance‐to‐tip with a slope of 0.38 (95% CI: 0.34–0.43, *P* < 0.001) in main stems and 0.27 (95% CI: 0.18–0.35, *P* < 0.001) in branches (Table 1; Fig. 3a,b). The distance‐to‐tip explained 60% of the variance in vessel median diameter in the main stem sample and 36% of the variance in the branch sample (Table 1). Both main stem and branch *D*_*H*_ also scaled with distance‐to‐tip with a slope of 0.39 (95% CI: 0.35–0.43, *P* < 0.001) and 0.39 (95% CI: 0.28–0.49, *P* < 0.001, Table 1; Fig. S2). While separating the widest vessels (upper quartile) and narrowest vessels (lower quartile), we found that both scaled with distance‐to‐tip (Table 1; Fig. S2).

**Table 1:** Parameters estimated by GLS or pGLS model on traits–distance‐to‐tip. Traits include diameters (D), density, and fraction. Upper and lower 95% of confidence intervals (CI), Akaike information criterion (AIC), Bayesian information criterion (BIC), and log‐likelihood (logLik) of the model were shown. When GLS is applied, Pagel's lambda and partial *R* ^2^ are not calculated. *P*‐values less than the statistical significance level (0.05) are reported in bold.

Organ	Tissue	Trait	Intercept	Slope	Lower 95% CI	Upper 95% CI	Slope *P*‐value	df	Pagel's λ	AIC	BIC	logLik	Partial *R* ^2^	*R* ^2^	Model type
Main stem	Sieve element	Hydraulic *D*	2.43	0.26	0.23	0.29	**< 0.001**	155		37.56	46.69	−15.78		0.68	gls
		Upper quantile *D*	2.49	0.26	0.23	0.29	**< 0.001**	155		40	49.13	−17		0.68	gls
		Median *D*	2.38	0.26	0.23	0.29	**< 0.001**	155		33.26	42.39	−13.63		0.69	gls
		Lower quantile *D*	2.28	0.26	0.23	0.29	**< 0.001**	155		31.92	41.05	−12.96		0.69	gls
		Density	7.34	−0.39	−0.47	−0.31	**< 0.001**	83		144.98	152.24	−69.49		0.52	gls
		Fraction	−1.78	0.08	0.01	0.14	**0.02**	83	0.41	78.58	88.26	−35.29	0.08	0.14	pgls
	Vessel	Hydraulic *D*	3.59	0.39	0.35	0.43	**< 0.001**	155		138.34	147.47	−66.17		0.71	gls
		Upper quantile *D*	3.66	0.38	0.33	0.42	**< 0.001**	155	0.37	155.75	167.93	−73.88	0.61	0.72	pgls
		Median *D*	3.44	0.38	0.34	0.43	**< 0.001**	155	0.36	160.27	172.41	−76.15	0.60	0.72	pgls
		Lower quantile *D*	3.14	0.38	0.34	0.42	**< 0.001**	155		167.88	177.01	−80.94		0.66	gls
		Density	4.9	−0.77	−0.94	−0.6	**< 0.001**	83	0.87	239.92	249.59	−116	0.46	0.75	pgls
		Fraction	−2.11	−0.1	−0.2	0.01	0.08	83	0.47	168.9	178.58	−80.45	0	0.26	pgls
Branch	Sieve element	Hydraulic *D*	2.47	0.19	0.14	0.23	**< 0.001**	73		9.46	16.33	−1.73		0.45	gls
		Upper quantile *D*	2.53	0.19	0.14	0.23	**< 0.001**	73		10.96	17.84	−2.48		0.45	gls
		Median *D*	2.43	0.19	0.14	0.24	**< 0.001**	73		13.8	20.67	−3.9		0.46	gls
		Lower quantile *D*	2.32	0.2	0.15	0.25	**< 0.001**	73		12.66	19.53	−3.33		0.48	gls
	Vessel	Hydraulic *D*	3.76	0.22	0.13	0.32	**< 0.001**	64	0.81	61.41	70.05	−26.71	0.17	0.47	gls
		Upper quantile *D*	3.81	0.22	0.13	0.32	**< 0.001**	64	0.61	67.85	76.48	−29.92	0.16	0.42	gls
		Median *D*	3.62	0.27	0.18	0.35	**< 0.001**	64		66.30	72.78	−30.15		0.36	gls
		Lower quantile *D*	3.32	0.24	0.16	0.32	**< 0.001**	64		56.52	63.00	−25.26		0.35	gls

Similarly, the median phloem sieve element diameter of both main stems and branch samples increased significantly with distance‐to‐tip, with a slope of 0.26 (95% CI: 0.23–0.29, *P* < 0.001) and 0.19 (95% CI: 0.14–0.24, *P* < 0.001), respectively (Table 1; Fig. 3a,b). The distance‐to‐tip explained 69% of the variance in sieve element median diameter in the main stem sample and 46% of the variance in the branch sample (Table 1). Within both main stems and branches, hydraulic diameter, upper quartile, and lower quartile of sieve element diameter were also scaled with distance‐to‐tip with similar scaling factors (Table 1; Fig. S2). Sieve element diameters and vessel diameters scaled with distance‐to‐tip with different scaling exponents in both main stems and branches (Table 1; Fig. S2). Hydraulic diameter, median, upper quartile, and lower quartile vessel diameters increased faster than the corresponding parameters for sieve elements. This difference is highlighted by the nonoverlapping 95% CIs of the slopes for vessels and sieve elements, indicating a steeper scaling relationship in vessels (Table 1).

### Vessel and sieve element densities decrease with distance‐to‐tip

In contrast to conduit size, both vessel and sieve element density decreased with distance‐to‐tip. Sieve element density (slope: −0.39, 95% CI: −0.47 to −0.31, *P* < 0.001) decreased more slowly than vessel density (slope: −0.77, 95% CI: −0.94 to −0.60, *P* < 0.001) and this difference was statistically significant (*P* < 0.001, Table 1; Fig. 3c).

Xylem vessel fraction was not significantly correlated with distance‐to‐tip (slope: −0.10, 95% CI: −0.20–0.01, *P* = 0.08, Table 1; Fig. 3d), and phloem sieve element fraction was positively correlated with distance‐to‐tip with a slope of 0.08 (95% CI: 0.01–0.14, *P* = 0.02, Table 1; Fig. 3d). However, distance‐to‐tip only explained a small proportion of the variation in sieve element lumen fraction (partial *R* ^*2*^ = 0.08, Table 1).

### Vessel and sieve element traits are weakly coordinated after detrending

**Fig. 4:**
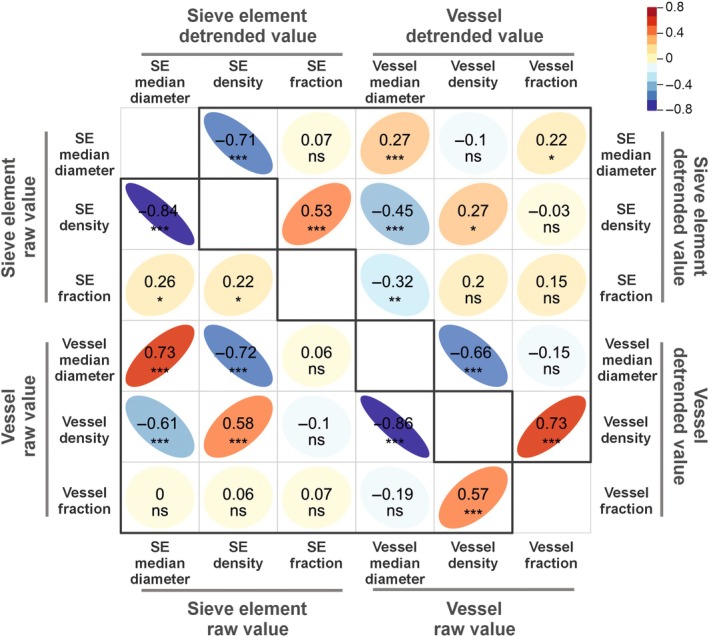
Pearson product–moment correlation between sieve element (SE) and vessel anatomical traits in main stem samples. The left triangle shows correlation between traits' raw value; the right triangle shows correlation between traits' detrended value, corrected after distance‐to‐tip. The color and the shape reflect the value of Pearson correlation coefficients. Significant code: ***, *P* < 0.001; **, *P* < 0.001; *, *P* < 0.5; ns, not significant, *P ≥* 0.05.

For between‐tissue correlation, when comparing the raw values across multiple species, both median diameter and density showed highly significant positive Pearson correlation coefficients between vessels and sieve elements (*R* = 0.73, *P* < 0.001, and *R* = 0.58, *P* < 0.001, respectively; Fig. 4). However, after removing the effect of distance‐to‐tip and comparing detrended trait values, these positive correlations remained statistically significant but were weaker (median diameter: *R* = 0.27, *P* < 0.001; density: *R* = 0.27, *P* < 0.001; Fig. 4). There was no significant correlation between vessel and sieve elements for fraction, whether in raw or detrended values (*R* = 0.07, *P* ≥ 0.05 and *R* = 0.15, *P* ≥ 0.05, respectively; Fig. 4).

For within‐tissue relationships, sieve element median diameter was negatively correlated with sieve element density for both raw and detrended values (*R* = −0.84, *P* < 0.001 and *R* = −0.71, *P* < 0.001, respectively; Fig. 4). Similarly, vessel median diameter was negatively correlated with density for both raw and detrended values (*R* = −0.86, *P* < 0.001 and *R* = −0.66, *P* < 0.001, respectively; Fig. 4). The raw value of sieve element fraction was statistically significant but weakly correlated with sieve element median diameter (*R* = 0.26, *P =* 0.02; Fig. 4) and density (*R* = 0.22, *P =* 0.04; Fig. 4). The raw value of vessel fraction was significantly correlated with vessel density (*R* = 0.57, *P <* 0.001; Fig. 4) but not with median diameter (*R* = −0.19, *P ≥* 0.05; Fig. 4). After detrending, the fraction of both sieve elements and vessels was significantly correlated with their density (*R* = 0.53, *P* < 0.001 and *R* = 0.73, *P* < 0.001, respectively; Fig. 4), but not significantly correlated with their median diameters (*R* = 0.07, *P ≥* 0.05 and *R* = −0.15, *P ≥* 0.05, respectively; Fig. 4).

### Climate impacts vessel and sieve element traits differently

The first three PCs explained 91.25% of the variance in all the 19 bioclim climate factors (Table S4). The first dimension (PC1) captured the overall variance across all factors (Table S4). The second dimension (PC2) primarily reflected overall temperature (Table S4), which was positively correlated with temperature‐related factors (annual temperature, maximum temperature of warmest month, mean temperature of driest quarter, and mean temperature of warmest quarter). The third dimension (PC3), reflecting water availability during the growing season, was negatively correlated with driest month precipitation and precipitation seasonality, and positively correlated with precipitation of the warmest quarter and mean temperature of the wettest quarter (Table S4).

**Fig. 5:**
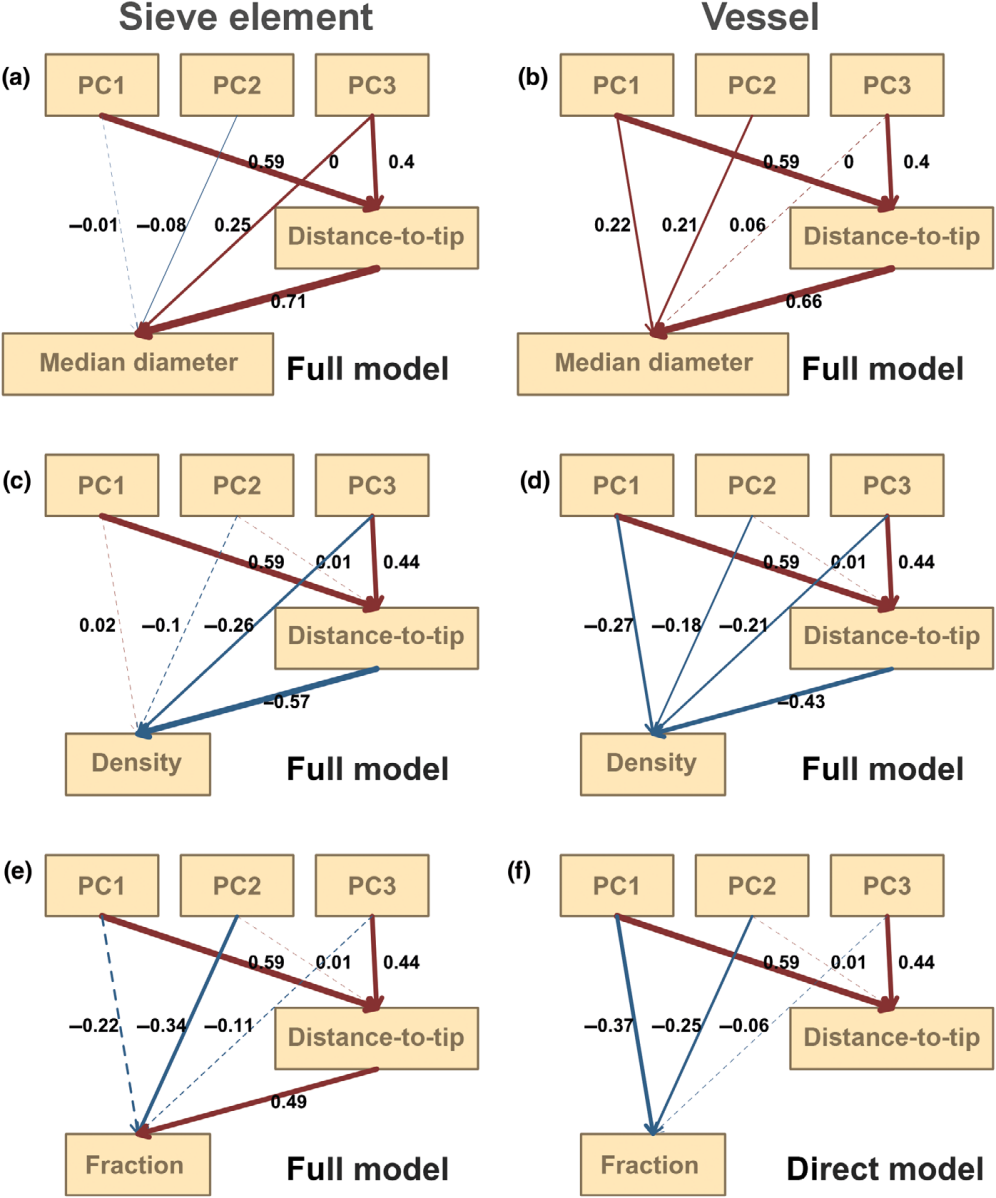
The best‐fit phylogenetic structural equation model for sieve element (a) median diameter, (c) density, and (e) fraction; and vessel (b) median diameter, (d) density, and (f) fraction in main stem samples. Arrows represent the influence of climate factors (PCs) or distance‐to‐tip on trait values. Red arrows show positive correlation, and blue arrows show negative correlation. The thickness of the arrow indicates the weight. Solid lines indicate 95% confidence intervals (CIs) do not overlap zero, and dashed lines indicate 95% CIs overlap zero.

Climate influenced both vessel and sieve element hydraulic diameter by indirect and direct paths as full models provided a better fit, primarily through indirect paths (Table 2; Fig. 5a,b). After removing the indirect influence of climate, vessel and sieve element *D*_*H*_ responded differently to climate factors: all the PCs had a direct impact on the vessel hydraulic diameter, while only PC3, which was mainly influenced by growing season water availability, had a direct effect on the sieve element hydraulic diameter (Table 2; Fig. 5a,b). Partial regression results also produced similar conclusions (Table S5). PC1 and PC2 were significantly positively correlated with the detrended vessel hydraulic diameter, median diameter, upper and lower quartiles. The three PCs of climate factor explained 7, 19, 15, and 17% of the variance, respectively (Table S5). The detrended value of sieve elements' diameter‐related traits only responded to PC3 (*R* ^2^: hydraulic diameter: 0.14; median diameter: 0.13, upper quartile: 0.14 and lower quartile: 0.12, Table S5).

**Table 2:** Phylogenetic corrected structural equation model parameter estimation. C‐statistic information criterion (CICc) and coefficients between two variables are shown in the table. When a direct model is applied, there are no estimated coefficients between *y* and *x*. When an indirect model is applied, there are no coefficients between PCs and *y*.

	Trait	Selected model	CICc	PC1~*x*	PC2~*x*	PC3~*x*	*y*~*x*	PC1~*y*	PC2~*y*	PC3~*y*
Sieve element	Hydraulic diameter	Full	40.78	0.59	0	0.4	0.71	−0.03	−0.08	0.26
	Diameter upper quantile	Full	40.78	0.59	0	0.4	0.72	−0.04	−0.09	0.25
	Diameter median	Full	40.78	0.59	0	0.4	0.71	−0.01	−0.08	0.25
	Diameter lower quantile	Full	40.78	0.59	0	0.4	0.69	0.01	−0.07	0.25
	Density	Full	36.15	0.59	0.01	0.44	−0.57	0.02	−0.1	−0.26
	Fraction	Full	36.15	0.59	0.01	0.44	0.49	−0.22	−0.34	−0.11
Vessel	Hydraulic diameter	Full	40.78	0.59	0	0.4	0.64	0.13	0.11	0.19
	Diameter upper quantile	Full	40.78	0.59	0	0.4	0.65	0.18	0.19	0.11
	Diameter median	Full	40.78	0.59	0	0.4	0.66	0.22	0.21	0.06
	Diameter lower quantile	Full	40.78	0.59	0	0.4	0.65	0.23	0.22	0.05
	Density	Full	36.16	0.59	0.01	0.44	−0.43	−0.27	−0.18	−0.21
	Fraction	Direct	33.60	0.59	0.01	0.44		−0.37	−0.25	−0.06

Climate mainly indirectly influenced sieve element density but impacted vessel density through both direct and indirect paths (Fig. 5c,d). Both pSEM and partial regression showed that PC1 and PC2 had a direct negative effect on vessel density, and PC1 and PC2 jointly explained 30% of the variance in detrended values after correcting for distance‐to‐tip (Table S5). Climate only directly influenced vessel fraction; PC1 and PC2 were both negatively correlated with vessel fraction, and they explained 18% of the detrended value variance (Fig. 5f; Table S5). The full model fitted better with sieve element fraction, but only PC2 significantly and negatively influenced sieve element fraction, and it explained 12% of the detrended value variance (Fig. 5e; Table S5).

## Discussion

Our results demonstrate that distance‐to‐tip is the primary determinant of sieve element diameter in the main stem sample, across 156 species from a wide taxonomic range in woody species. Furthermore, we demonstrate that the scaling of sieve element traits along the stem does not simply mirror the scaling of vessel traits. In addition, climate plays an explanatory role in the diameter of vessels and sieve elements. Unlike vessel diameters, which mainly vary along a temperature gradient, sieve element diameters change according to water availability when distance‐to‐tip is considered.

One of our most striking findings was that, contrary to the view that sieve elements are mirroring vessels due to a strong connectivity between the xylem and the phloem (Savage *et al*., 2017), we found that the diameters of sieve elements and vessels do not scale linearly, as previously reported in *Fraxinus excelsior* L. and *Salix eleagnos* Scop. (Petit & Crivellaro, 2014). From the tip of the stem to the base of the plant, vessels increased in size faster than sieve elements. Auxin concentration is associated with cell size; different plant tissues exhibit varied sensitivities to auxin (Tang *et al*., 2025), which may lead to different responses in xylem and phloem tissue, even though they arise from the same cambium. Additionally, as sieve elements are living cells with thin primary walls, they might be more size‐constrained than vessels that are dead cells at maturity with thick secondary walls. These constraints could be both biomechanical and functional, as sieve elements in their mature state are no longer autonomous cells and are strongly dependent on the support of the neighboring companion cells. For example, some proteins for metabolic activities in sieve elements were transported from companion cells (Knoblauch *et al*., 2018; Matilla, 2023). This structural limitation probably translates into a maximal size threshold over which the capacity for the exchange of proteins with the companion cells becomes limiting (Oparka & Turgeon, 1999).

Despite the differences in their respective scaling factors, our results confirm the expected concurrent widening patterns of xylem vessels and sieve elements with increasing distance‐to‐tip, extending the patterns previously reported in taxonomically narrower groups (Petit & Crivellaro, 2014; Savage *et al*., 2017; Clerx *et al*., 2020). The allometric relationships of the vascular system in plants have been extensively studied, both theoretically and empirically (West *et al*., 1999; Hölttä *et al*., 2013; Tyree & Zimmermann, 2013). As defined by Huxley (1932), as cited Enquist (2002), any organ, including both the sizes of xylem conduits and phloem sieve elements (*Y*), should also follow the allometric equation: *Y* = *Y*_0_ *M* ^b^, where *Y*_0_ is a constant, *M* is a size‐related parameter, and b is an allometric exponent. This concurrent widening of vessels and sieve elements with increasing distance‐to‐tip may be due to a functional constraint. The efficiency of sap conduction is maximized as both elements' sizes increase further from the tip (Knoblauch *et al*., 2016; Savage *et al*., 2017). The mechanisms underlying this pattern may involve hormonal and developmental regulation, as both types of cells are regulated by a similar pathway (Aloni & Zimmermann, 1983; Ali *et al*., 2023). Alongside the effect of the turgor gradient, the widening may also result from a longitudinal auxin gradient in the cambium. Aloni & Zimmermann (1983) suggested that lower auxin levels at the plant base slow xylem cell differentiation and extend the expansion phase, promoting elongation (Bhalerao & Bennett, 2003). The duration of this phase correlates positively with xylem lumen size (Anfodillo *et al*., 2012). The same mechanism could also affect the phloem sieve element, but that question is beyond the scope of the present study. Therefore, to understand the processes creating this scaling, further studies are needed to investigate hormone levels in secondary phloem along the stem and whether cambium cell width itself scales with distance‐to‐tip.

Our study underscores the necessity of considering distance‐to‐tip when analyzing anatomical variations linked to the environment in woody plants. Since the distance‐to‐tip primarily influences vessel and sieve element size and density, failing to do so may lead to misleading conclusions due to size‐related sampling biases. Structural equation models show that the effects of the environment on conduit diameter and density are largely indirect; that is, they have a direct influence on plant size, which translates into different distance‐to‐tip and consequently affects the size and density of conduits. The correlation of water availability with xylem vessel size (Sonsin *et al*., 2012; Warwick *et al*., 2017; García‐Cervigón *et al*., 2018) is frequently interpreted as a causal relationship when it could simply reflect hidden correlation with stem length (Olson *et al*., 2014; Fajardo *et al*., 2020). Our results suggest that a similar bias may apply to sieve elements if the distance‐to‐tip is not considered. This is consistent with the relationship between smaller sieve elements in drier environments found in *Fagus sylvatica* L. (Dannoura *et al*., 2019), but this relationship was not significant when the tree height was considered (Liesche *et al*., 2017).

Controlling for distance‐to‐tip also reduced the interspecific correlation between sieve element and vessel diameter, indicating that phloem traits may respond differently to the environmental factors compared with xylem traits, and thus provide complementary insights into xylem traits in understanding species–environment relationships. A previous study showed that sieve element properties were strongly linked to vessel properties but were either not linked or only weakly linked to the environment (Tang *et al*., 2022). Our results suggest that the size or density of sieve elements and vessels across species respond differently to the climatic factors after acknowledging their main source of variation, that is their location along the stem, which might result from the very different physiological roles of both structures. In our results, detrended vessel diameter is positively correlated to temperature (PC2), in line with previous studies across angiosperms (Olson *et al*., 2014, 2018, 2021; Morris *et al*., 2018), whereas Kašpar *et al*. (2019) reported that no difference within some conifers species. By contrast, species‐specific sieve element diameter was positively correlated only with the typical water availability during the growing season (PC3). Our results did not support the hypothesis of Sevanto (2018), who suggested that sieve elements would have wider diameters to compensate for higher viscosity at low water availability, but aligned with the prediction of Salmon *et al*. (2019), who suggested that less water availability during the growing season may decrease the water potential in the xylem transport, thus influencing the phloem turgor and ultimately sieve element cell expansion. Our analyses showed that sieve element diameters did not follow the same temperature gradient patterns as vessel diameters. As freeze–thaw‐induced embolism is associated with larger vessel diameters (Zhang *et al*., 2024), selection might favor small vessels in cold environments, while warmer environments are associated with higher transpiration and, thus, the need for more efficient water transport. Similar selective pressure might not apply to sieve elements. Indeed, unlike the xylem conduits (Hacke & Sperry, 2001; Konrad *et al*., 2019), sieve elements do not risk freeze–thaw‐induced embolism, as phloem water potential remains positive (Hall & Minchin, 2013), removing the benefit of smaller conduits in cooler climates.

Building on these findings, methods that explicitly account for allometry effects are essential for accurately assessing how environmental factors influence species traits. Kašpar *et al*. (2019) proposed standardizing vessel diameter by height using a power relationship with a fixed exponent. Olson *et al*. (2014, 2021) proposed calculating the residuals of the anatomical traits after removing the part of the variation explained by the distance‐to‐tip, an approach we adopted as detrending. Detrended values allow us to appropriately evaluate the variance attributed to external (environment) and internal (allometry) explanatory variables, avoiding collinearity and providing flexibility in predicted scaling factors. As an alternative, our study showed that structural equation models provided a reliable method and a broader perspective to study how the environment shapes the size of conduits through different pathways, by including both external effects and internal constraints in the same model. This analysis allowed us to compare the relative importance of these two paths and avoid confounding effects, especially where trait–distance relationships, such as for lumen fraction, are unclear.

While conduit diameter and density variations across species were mainly explained by distance‐to‐tip, conduit fraction variation was influenced more by the environment. The influence of distance‐to‐tip was minor because conduit fractions remained largely constant along the length of the main stem. Previous studies have proposed a trade‐off between vessel size and number (Sperry *et al*., 2008; Olson *et al*., 2020). Sperry *et al*. (2008) suggested a ‘packing limit’ for the combination of density and diameter of vessels. According to that suggestion, plants tend to maintain the same area, volume, or conductance along the axes (Sopp & Valbuena, 2023). Our study demonstrated that this ‘conduit area‐preservation’ pattern along the stem applies to phloem sieve elements, likely due to the limited space in the conducting phloem. In colder environments, despite smaller vessel size, an increase in vessel density leads to a greater total lumen area of both vessels and sieve elements. Both vessel fraction and sieve element fraction were negatively correlated with temperature‐driven PC2, but vessel fraction was also correlated with PC1. Zheng *et al*. (2019) found that vessel lumen fractions decreased with mean annual temperature in angiosperm woody species and suggested that higher vessel tissue fractions may offset reduced conductivity due to smaller vessel diameters in colder climates (Schreiber *et al*., 2015; Plavcová *et al*., 2024). Sieve element fraction adjusts to temperature with a similar pattern as vessel fraction, but climate explains much less variance. Interestingly, vessel fraction and sieve element fraction were not correlated in our study. How plants organize the fraction and quantity of sieve elements to couple the transport capacity of xylem and ultimately match photosynthetic production and water–carbon balance of the plant should be studied by considering conductive phloem width and sapwood area in the future.

Like previous studies on vessel diameters across species (Olson *et al*., 2014, 2021), our study focuses on the link between general climate and anatomical features. We acknowledge that such studies might not fully capture the growth‐limiting factors and the critical periods for xylem or phloem development, which can vary with climates. Furthermore, our study cannot separate individual environmental responses from species‐level adaptations, as most of the samples are from one location. Nonetheless, our novel findings open a new field of investigation into phloem anatomical traits, and encourage further study of intra‐specific phloem trait variation, as well as the use of common garden experiments, to identify environmental factors that influence sieve element formation and expansion with more detailed inter‐annual information.

### Conclusion

In conclusion, our study highlights distance‐to‐tip as a dominant driver of conduit anatomy across woody species, shaping both xylem vessel and phloem sieve element size, and therefore must be accounted for in anatomical studies. Although both conduits widen basipetally along the stem, xylem vessels increase in size faster than phloem sieve elements from the tip to the base, and their environmental responses diverge. Vessel diameter is positively correlated to temperature, whereas sieve element diameter reflects water availability. These findings challenge the assumption that phloem traits merely mirror xylem traits. We separate environmental signals from internal scaling effects by applying detrending and structural equation models. This approach proved a powerful tool to refine our understanding of plant vascular adaptation and invites further research into phloem function, plasticity, and developmental controls.

## Competing interests

None declared.

## Author contributions

YW, YS, JL and AC conceived the ideas. YW designed the study. YS secured the funding. JD, JA, DC, AC, AD, ZF, PF, JG, PH, SI, AM, CBKR, ET and ST collected the samples and offered cross‐section slides. FF and OM provided the phytogenic trees. YW measured the anatomical traits and analyzed the data. YW wrote the original draft of the manuscript with help from JL and YS. All authors reviewed and edited the manuscript.

## Disclaimer

The New Phytologist Foundation remains neutral with regard to jurisdictional claims in maps and in any institutional affiliations.

## Supporting information


**Fig. S1** Example of the anatomical cuttings on phloem sieve elements.
**Fig**. **S2** Scaling factor of main stem samples with the distance‐to‐tip.
**Table S1** Sample information.
**Table S2** Anatomical slides preparation methods.
**Table S3** Linear regression model on samples from different laboratories.
**Table S4** PC loadings of environmental factors.
**Table S5** Partial regressions on response of trait to environmental factors.
Please note: Wiley is not responsible for the content or functionality of any Supporting Information supplied by the authors. Any queries (other than missing material) should be directed to the *New Phytologist* Central Office.

## Data Availability

The numerical data that support the findings of this study are openly available in the Zenodo repository at doi: 10.5281/zenodo.17037641. Original cuttings are available upon request.
