# Supplementary material for: Physical constraints and environmental factors shape phloem anatomical traits in woody angiosperm species
Source: New Phytol. 2025 Sep 15;248(5):2316–30. doi: 10.1111/nph.70578 (PMC12589700; doi:10.1111/nph.70578)
Supplement: Supplementary file 1 — Fig. S1 Example of the anatomical cuttings on phloem sieve elements. Fig. S2 Scaling factor of main stem samples with the distance‐to‐tip. Table S1 Sample information. Table S2 Anatomical slides preparation methods. Table S3 Linear regression model on samples from different laboratories. Table S4 PC loadings of environmental factors. Table S5 Partial regressions on response of trait to environmental factors. Please note: Wiley is not responsible for the content or functionality of any Supporting Information supplied by the authors. Any queries (other than missing material) should be directed to the New Phytologist Central Office. [file NPH-248-2316-s001.pdf]

New Phytologist

New Phytologist Supporting Information

Article title: Physical constraints and environmental factors shape phloem anatomical traits in woody plant species
Authors: Yan Wang, Johannes Liesche, Alan Crivellaro, Jiří Doležal, Jan Altman, Donato Chiatante, Anastazija Dimitrova, Zexin Fan, Peili Fu, Felix Forest, Jozica Gričar, Patrick Heuret, Sandrine Isnard, Olivier Maurin, Antonio Montagnoli, Cyrille B. K. Rathgeber, Enkhchimeg Tsedensodnom, Santiago Trueba, Yann Salmon
Article acceptance date: 30 August 2025
The following Supporting Information is available for this article:

Fig. S1 Example of the anatomical cuttings on phloem sieve elements.

Fig. S2 Scaling factor of main stem samples with the distance-to-tip.

Table S1 Sample information

Table S2 Anatomical slides preparing methods

Table S3 Linear regression model on samples from different laboratories

Table S4 PC loadings of environment fact

Table S5 Partial regressions on response of trait to environment factors

Fig. S1 Example of the anatomical cuttings on phloem sieve elements. The stars indicate sieve elements with or without company cells (yellow arrow). Scale bars: 100 µm. (a) *Quercus pubescens* Willd. Microcores samples, embedded in paraffin, sectioned with rotary microtome, and processed at Slovenian Forestry Institut. Sieve elements are often associated with companion cells. The arrangement of the sievee, Slovenia elements is irregular, but their size and shape differ from those of axial parenchyma cells. (b) *Myrtus communis* L. Disk samples, sectioned with sliding microtome, and processed at University of Padova, Italy. Sieve elements are not always associated with companion cells and their size does not differ from the adjacent axial parenchyma cell. However, their shape is distinct. Additionally, axial parenchyma cells are arranged in parallel. (c) *Anthonotha ferruginea* (Harms) J.Léonard. Microcores samples, sectioned with sledge microtome, and processed at Institute of Botany of the Czech Academy of Sciences, Trebon, Czech. The size and shape of the sieve element differ from those of axial parenchyma cells. In some cases, sieve plates are visible (purple arrows). (d) *Melia toosendan* L.

Microcores samples, buried in paraffin, sectioned with rotary microtome, and processed at Xishuangbanna Tropical Botanical Garden, China. Sieve elements are not always associated with companion cells, and their arrangement is irregular. However, the size and shape of the sieve element differ from those of axial parenchyma cells. Sometimes the sieve plates are visible (purple arrows).

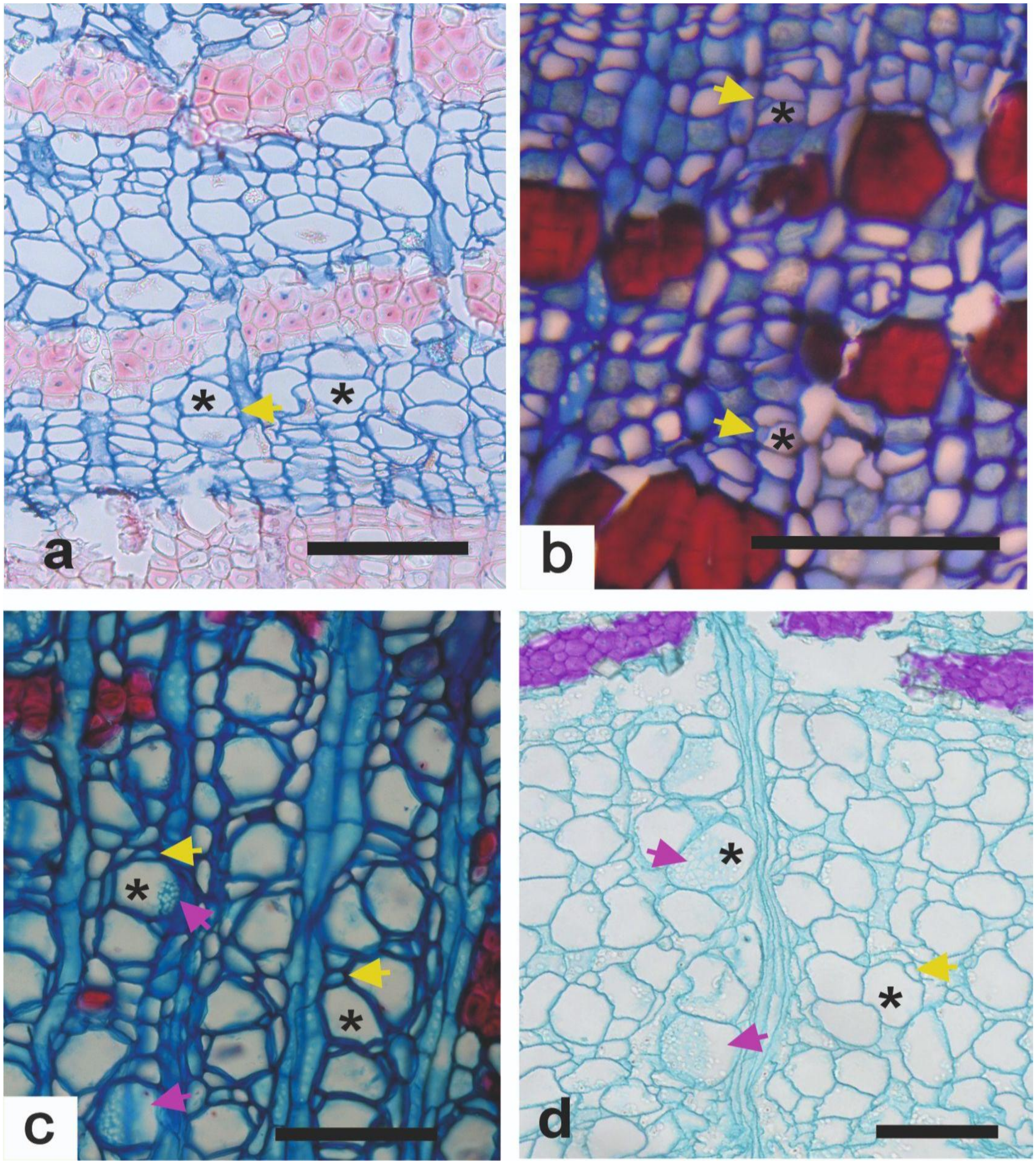

Fig. S2 Scaling factor of (a) hydraulic diameter, (b) upper quantile diameter in stem sample, (c) lower quartile diameter with distance-to-tip in main stem. The exponents (b) were shown in the figure. Solid line: slope b is significantly different from zero ($P < 0.05$). Dashed line: slope b is not significantly different from zero ($P \geqslant 0.05$).

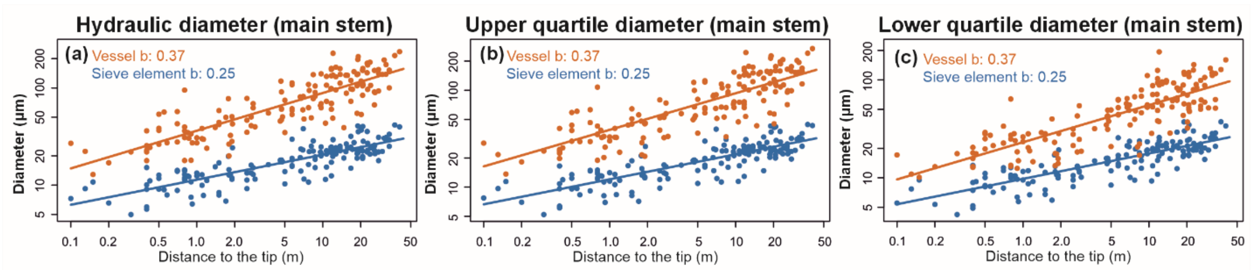

Table S1 Sample information. Species names, their correspondence name on the phylogenetic tree for pgls, sample organ type, sample size and preparing methods were shown. When the method is not published, only the processing laboratory is shown.

Species	Family	Species signed for pgls	Organ	Country	Climate	Individuals	Method
Ballota integrifolia	Lamiaceae	*Acanthoprasium integrifolium*	Tr, Br	Cyprus	mediterranean	1	Crivellaro *et al.* 2012
Afrostyrax lepidophyllus	Huaceae	*Afrostyrax lepidophyllus*	Tr	Cameroon	wet tropical	1	Plavcová *et al.* 2024
Alangium chinense	Cornaceae	*Alangium chinense*	Tr	Cameroon	seasonally dry tropical	1	Plavcová *et al.* 2024
Albizia zygia	Fabaceae	*Albizia zygia*	Tr	Cameroon	seasonally dry tropical	2	Plavcová *et al.* 2024
Alhagi graegorum	Fabaceae	*Alhagi graecorum*	Tr	Cyprus	mediterranean	1	Crivellaro *et al.* 2012
Allophylus bullatus	Sapindaceae	*Allophylus bullatus*	Tr	Cameroon	wet tropical	1	Plavcová *et al.* 2024
Pouteria pierrei	Sapotaceae	*Aningeria pierrei*	Tr	Cameroon	wet tropical	1	Plavcová *et al.* 2024
Annickia chlorantha	Annonaceae	*Annickia chlorantha*	Tr	Cameroon	wet tropical	1	Plavcová *et al.* 2024
Anthonotha ferruginea	Fabaceae	*Anthonotha ferruginea*	Tr	Cameroon	wet tropical	2	Plavcová *et al.* 2024
Anthonotha macrophylla	Fabaceae	*Anthonotha macrophylla*	Tr	Cameroon	wet tropical	1	Plavcová *et al.* 2024
Antidesma membranaceum	Phyllanthaceae	*Antidesma membranaceum*	Tr	Cameroon	wet tropical	1	Plavcová *et al.* 2024
Arbutus unedo x andrachnoides	Ericaceae	*Arbutus andrachne*	Tr, Br	Cyprus	mediterranean	1	Crivellaro *et al.* 2012
Arbutus unedo	Ericaceae	*Arbutus unedo*	Tr, Br	Cyprus	temperate	1	Crivellaro *et al.* 2012
Sorbus aria	Rosaceae	*Aria edulis*	Tr, Br	Cyprus	temperate	1	Crivellaro *et al.* 2012
Asperula cypria	Rubiaceae	*Asperula cypria*	Tr, Br	Cyprus	mediterranean	1	Crivellaro *et al.* 2012
Astropanax mannii	Araliaceae	*Astropanax mannii*	Tr	Cameroon	wet tropical	1	Plavcová *et al.* 2024
Autranella congolensis	Sapotaceae	*Autranella congolensis*	Tr	Cameroon	wet tropical	1	Plavcová *et al.* 2024
Barringtonia racemosa	Lecythidaceae	*Barringtonia racemosa*	Tr	China	wet tropical	3	XTBG, China
Bauhinia variegata	Fabaceae	*Bauhinia variegata*	Tr	Cyprus	seasonally dry tropical	1	Crivellaro *et al.* 2012
Beilschmiedia manii	Lauraceae	*Beilschmiedia mannii*	Tr	Cameroon	wet tropical	1	Plavcová *et al.* 2024
Berberis cretica	Berberidaceae	*Berberis cretica*	Tr, Br	Cyprus	mediterranean	1	Crivellaro *et al.* 2012
Bocoa prouacensis	Fabaceae	*Bocoa prouacensis*	Br	French Guiana	wet tropical	1	Levionnois *et al.* 2021
Broussonetia papyrifera	Moraceae	*Broussonetia papyrifera*	Tr	China	temperate	5	XTBG, China
Calycotome villosa	Fabaceae	*Calicotome villosa*	Tr, Br	Cyprus	mediterranean	1	Crivellaro *et al.* 2012
Caloncoba glauca	Achariaceae	*Caloncoba glauca*	Tr	Cameroon	wet tropical	3	Plavcová *et al.* 2024

Ceiba pentandra	Malvaceae	*Ceiba pentandra*	Tr	Cameroon	seasonally dry tropical	1	Plavcová *et al.* 2024
Celtis australis	Cannabaceae	*Celtis australis*	Tr, Br	Cyprus	mediterranean	1	Crivellaro *et al.* 2012
Celtis tournefortii	Cannabaceae	*Celtis tournefortii*	Tr, Br	Cyprus	mediterranean	1	Crivellaro *et al.* 2012
Chaetocarpus schomburgkianus	Peraceae	*Chaetocarpus schomburgkianus*	Br	French Guiana	wet tropical	1	Levionnois *et al*. 2021
Chrysophyllum sanguinolentum	Sapotaceae	*Chrysophyllum sanguinolentum*	Br	French Guiana	wet tropical	2	Levionnois *et al*. 2021
Chukrasia tabularis	Meliaceae	*Chukrasia tabularis*	Tr	China	wet tropical	3	XTBG, China
Chytranthus talbotii	Sapindaceae	*Chytranthus talbotii*	Tr	Cameroon	wet tropical	1	Plavcová *et al.* 2024
Cistus creticus	Cistaceae	*Cistus creticus*	Tr, Br	Cyprus	mediterranean	1	Crivellaro *et al*. 2012
Cistus parviflorus x monspeliensis	Cistaceae	*Cistus parviflorus*	Tr, Br	Cyprus	mediterranean	1	Crivellaro *et al.* 2012
Cistus salviifolius	Cistaceae	*Cistus salviifolius*	Tr, Br	Cyprus	mediterranean	1	Crivellaro *et al*. 2012
Citrus maxima	Rutaceae	*Citrus maxima*	Tr, Br	Cyprus	wet tropical	1	Crivellaro *et al.* 2012
Clausena anisata	Rutaceae	*Clausena anisata*	Tr	Cameroon	seasonally dry tropical	2	Plavcová *et al.* 2024
Coelocaryon preussii	Myristicaceae	*Coelocaryon preussii*	Tr	Cameroon	wet tropical	1	Plavcová *et al.* 2024
Cola digitata	Malvaceae	*Cola digitata*	Tr	Cameroon	wet tropical	1	Plavcová *et al.* 2024
Cola rostrata	Malvaceae	*Cola rostrata*	Tr	Cameroon	wet tropical	1	Plavcová *et al.* 2024
Cordia africana	Boraginaceae	*Cordia africana*	Tr	Cameroon	seasonally dry tropical	1	Plavcová *et al*. 2024
Cordia aurantiaca	Boraginaceae	*Cordia aurantiaca*	Tr	Cameroon	wet tropical	1	Plavcová *et al.* 2024
Corylus avellana	Betulaceae	*Corylus avellana*	Br	Swiss	temperate	1	Crivellaro *et al*. 2012
Cotoneaster racemiflorus	Rosaceae	*Cotoneaster racemiflorus*	Br	Italy	temperate	1	Crivellaro *et al.* 2012
Crataegus azarolus	Rosaceae	*Crataegus azarolus*	Tr, Br	Cyprus	mediterranean	1	Crivellaro *et al*. 2012
Crataegus monogyna	Rosaceae	*Crataegus monogyna*	Tr, Br	Cyprus	temperate	1	Crivellaro *et al*. 2012
Croton macrostachyus	Euphorbiaceae	*Croton macrostachyus*	Tr	Cameroon	seasonally dry tropical	1	Plavcová *et al.* 2024
Crudia gabonensis	Fabaceae	*Crudia gabonensis*	Tr	Cameroon	wet tropical	1	Plavcová *et al.* 2024
Cytrogonone argentea	Euphorbiaceae	*Cyrtogonone argentea*	Tr	Cameroon	wet tropical	1	Plavcová *et al.* 2024
Dicorynia guianensis	Fabaceae	*Dicorynia guianensis*	Br	French_Guiana	wet tropical	1	Levionnois *et al*. 2021
Diogoa zenkeri	Olacaceae	*Diogoa zenkeri*	Tr	Cameroon	wet tropical	3	Plavcová *et al.* 2024
Diospyros bipindensis	Ebenaceae	*Diospyros bipindensis*	Tr	Cameroon	wet tropical	1	Plavcová *et al*. 2024
Discoglypremma caloneura	Euphorbiaceae	*Discoglypremna caloneura*	Tr	Cameroon	wet tropical	1	Plavcová *et al*. 2024
Drypetes staudtii	Putranjivaceae	*Drypetes staudtii*	Tr	Cameroon	wet tropical	1	Plavcová *et al*. 2024
Entandrophragma utile	Meliaceae	*Entandrophragma utile*	Tr	Cameroon	wet tropical	1	Plavcová *et al.* 2024

Eperua falcata	Fabaceae	*Eperua falcata*	Br	French Guiana	wet tropical	1	Levionnois *et al*. 2021
Eperua grandiflora	Fabaceae	*Eperua grandiflora*	Br	French Guiana	wet tropical	1	Levionnois *et al.* 2021
Erica sicula	Ericaceae	*Erica sicula*	Tr, Br	Cyprus	mediterranean	1	Crivellaro *et al*. 2012
Eschweilera coriacea	Lecythidaceae	*Eschweilera coriacea*	Br	French Guiana	wet tropical	1	Levionnois *et al*. 2021
Eschweilera sagotiana	Lecythidaceae	*Eschweilera sagotiana*	Br	French Guiana	wet tropical	2	Levionnois *et al*. 2021
Eucalyptus salubris	Myrtaceae	*Eucalyptus salubris*	Br	Cyprus	dry shrubland	1	Crivellaro *et al*. 2012
Eucalyptus torquata	Myrtaceae	*Eucalyptus torquata*	Tr	Cyprus	dry shrubland	1	Crivellaro *et al*. 2012
Euphorbia thompsonii	Euphorbiaceae	*Euphorbia thompsonii*	Tr	Cyprus	mediterranean	1	Crivellaro *et al*. 2012
Fagus sylvatica	Fagaceae	*Fagus sylvatica*	Tr	France	temperate	1	Adikurnia and Rathgeber 2025
Ficus carica	Moraceae	*Ficus carica*	Tr, Br	Cyprus	temperate	1	Crivellaro *et al*. 2012
Ficus chlamydocarpa	Moraceae	*Ficus chlamydocarpa*	Tr	Cameroon	wet tropical	1	Plavcová *et al*. 2024
Ficus exasperata	Moraceae	*Ficus exasperata*	Tr	Cameroon	seasonally dry tropical	1	Plavcová *et al*. 2024
Ficus sur	Moraceae	*Ficus sur*	Tr	Cameroon	seasonally dry tropical	1	Plavcová *et al*. 2024
Flacourtia indica	Salicaceae	*Flacourtia indica*	Tr	Cameroon	seasonally dry tropical	2	Plavcová *et al*. 2024
Fraxinus ornus	Oleaceae	*Fraxinus ornus*	Tr	Slovenia	temperate	3	Gricar and Prislan 2022
Fumana arabica	Cistaceae	*Fumana arabica*	Tr	Cyprus	mediterranean	1	Crivellaro *et al*. 2012
Funtomia elastica	Apocynaceae	*Funtumia elastica*	Tr	Cameroon	seasonally dry tropical	1	Plavcová *et al*. 2024
Chrysophyllum africanum	Sapotaceae	*Gambeya africana*	Tr	Cameroon	wet tropical	2	Plavcová *et al*. 2024
Garuga floribunda	Burseraceae	*Garuga floribunda*	Tr	China	wet tropical	3	XTBG, China
Glyphaea brevis	Malvaceae	*Glyphaea brevis*	Tr	Cameroon	seasonally dry tropical	1	Plavcová *et al*. 2024
Gmelina arborea	Lamiaceae	*Gmelina arborea*	Tr	China	seasonally dry tropical	4	XTBG, China
Goupia glabra	Goupiaceae	*Goupia glabra*	Br	French Guiana	wet tropical	2	Levionnois *et al*. 2021
Greenwayodendron suaveolens	Annonaceae	*Greenwayodendron suaveolens*	Tr	Cameroon	wet tropical	1	Plavcová *et al*. 2024
Gustavia hexapetala	Lecythidaceae	*Gustavia hexapetala*	Br	French Guiana	wet tropical	1	Levionnois *et al*. 2021
Harungana madagascariensis	Hypericaceae	*Harungana madagascariensis*	Tr	Cameroon	wet tropical	1	Plavcová *et al*. 2024
Hevea brasiliensis	Euphorbiaceae	*Hevea brasiliensis*	Tr	China	wet tropical	2	XTBG, China
Homalium letestui	Salicaceae	*Homalium letestui*	Tr	Cameroon	wet tropical	1	Plavcová *et al*. 2024
Ilex grandiflora	Aquifoliaceae	*Ilex grandiflora*	Tr	Cameroon	wet tropical	1	Plavcová *et al*. 2024

Ilex mitis	Aquifoliaceae	*Ilex mitis*	Tr	Cameroon	wet tropical	1	Plavcová *et al*. 2024
Ixora nematopoda	Rubiaceae	*Ixora nematopoda*	Tr	Cameroon	wet tropical	1	Plavcová *et al*. 2024
Juglans regia	Juglandaceae	*Juglans regia*	Br	Cyprus	temperate	1	Crivellaro *et al*. 2012
Kigelia africana	Bignoniaceae	*Kigelia africana*	Tr	Cameroon	seasonally dry tropical	1	Plavcová *et al*. 2024
Klainedoxa gabonensis	Irvingiaceae	*Klainedoxa gabonensis*	Tr	Cameroon	wet tropical	1	Plavcová *et al*. 2024
Lannea welwitschii	Anacardiaceae	*Lannea welwitschii*	Tr	Cameroon	wet tropical	1	Plavcová *et al*. 2024
Lecythis persistens	Lecythidaceae	*Lecythis persistens*	Br	French Guiana	wet tropical	3	Levionnois *et al*. 2021
Lecythis poiteaui	Lecythidaceae	*Lecythis poiteaui*	Br	French Guiana	wet tropical	2	Levionnois *et al*. 2021
Licania membranacea	Chrysobalanaceae	*Licania membranacea*	Br	French Guiana	wet tropical	2	Levionnois *et al*. 2021
Lycium ferocissimum	Solanaceae	*Lycium ferocissimum*	Tr, Br	Cyprus	mediterranean	1	Crivellaro *et al*. 2012
Maesa lanceolata	Primulaceae	*Maesa lanceolata*	Tr	Cameroon	seasonally dry tropical	1	Plavcová *et al*. 2024
Trevia nudiflora	Euphorbiaceae	*Mallotus nudiflorus*	Tr	China	wet tropical	2	XTBG, China
Margaritaria discoidea	Phyllanthaceae	*Margaritaria discoidea*	Tr	Cameroon	seasonally dry tropical	2	Plavcová *et al*. 2024
Melaleuca almillaris	Myrtaceae	*Melaleuca armillaris*	Tr, Br	Cyprus	subtropical	1	Crivellaro *et al*. 2012
Melia toosendan	Meliaceae	*Melia azedarach*	Tr	China	seasonally dry tropical	4	XTBG, China
Grewia coriacea	Malvaceae	*Microcos coriacea*	Tr	Cameroon	wet tropical	1	Plavcová *et al*. 2024
Moronobea coccinea	Clusiaceae	*Moronobea coccinea*	Br	French Guiana	wet tropical	1	Levionnois *et al*. 2021
Morus alba	Moraceae	*Morus alba*	Tr, Br	Cyprus	temperate	1	Crivellaro *et al*. 2012
Morus nigra	Moraceae	*Morus nigra*	Br	Italy	temperate	1	Crivellaro *et al*. 2012
Musanga cecropioides	Urticaceae	*Musanga cecropioides*	Tr	Cameroon	wet tropical	1	Plavcová *et al*. 2024
Myrtus communis	Myrtaceae	*Myrtus communis*	Tr, Br	Cyprus	mediterranean	1	Crivellaro *et al*. 2012
Neuboutonia mannii	Euphorbiaceae	*Neoboutonia mannii*	Tr	Cameroon	wet tropical	2	Plavcová *et al*. 2024
Nerium oleander	Apocynaceae	*Nerium oleander*	Tr	Cyprus	subtropical	1	Crivellaro *et al*. 2012
Nuxia congesta	Stilbaceae	*Nuxia congesta*	Tr	Cameroon	seasonally dry tropical	1	Plavcová *et al*. 2024
Octoknema affinis	Olacaceae	*Octoknema affinis*	Tr	Cameroon	wet tropical	1	Plavcová *et al*. 2024
Odontites cypria	Orobanchaceae	*Odontites linkii*	Tr	Cyprus	mediterranean	1	Crivellaro *et al*. 2012
Olea capensis	Oleaceae	*Olea capensis*	Tr	Cameroon	subtropical	1	Plavcová *et al*. 2024
Olea europaea	Oleaceae	*Olea europaea*	Tr, Br	Cyprus	subtropical	1	Crivellaro *et al*. 2012
Onosma fruticosa	Boraginaceae	*Onosma fruticosa*	Tr, Br	Cyprus	mediterranean	1	Crivellaro *et al*. 2012
Ostrya carpinifolia	Betulaceae	*Ostrya carpinifolia*	Tr	Slovenia	temperate	3	Gricar and Prislan 2022

Oubanguia africana	Lecythidaceae	*Oubanguia africana*	Tr	Cameroon	wet tropical	1	Plavcová *et al*. 2024
Oubanguia alata	Lecythidaceae	*Oubanguia alata*	Tr	Cameroon	wet tropical	1	Plavcová *et al*. 2024
Phillyrea latifolia	Oleaceae	*Phillyrea latifolia*	Tr, Br	Cyprus	mediterranean	1	Crivellaro *et al*. 2012
Phlomis brevibracteata	Lamiaceae	*Phlomis brevibracteata*	Tr, Br	Cyprus	mediterranean	1	Crivellaro *et al*. 2012
Phlomis lunariifolia	Lamiaceae	*Phlomis lunariifolia*	Tr, Br	Cyprus	mediterranean	1	Crivellaro *et al*. 2012
Pistacia atlantica	Anacardiaceae	*Pistacia atlantica*	Tr, Br	Cyprus	subtropical	1	Crivellaro *et al*. 2012
Pittosporum viridiflorum	Pittosporaceae	*Pittosporum viridiflorum*	Tr	Cameroon	seasonally dry tropical	1	Plavcová *et al*. 2024
Platanus orientalis	Platanaceae	*Platanus orientalis*	Tr, Br	Cyprus	temperate	1	Crivellaro *et al*. 2012
Putoria calabrica	Rubiaceae	*Plocama calabrica*	Tr	Cyprus	temperate	1	Crivellaro *et al*. 2012
Pometia pinnata	Sapindaceae	*Pometia pinnata*	Tr	China	wet tropical	4	XTBG, China
Populus x sibirica	Salicaceae	*Populus balsamifera*	Tr	Mongolia	temperate	3	Dimitrova *et al*. 2024
Pradosia cochlearia	Sapotaceae	*Pradosia cochlearia*	Br	French Guiana	wet tropical	2	Levionnois *et al*. 2021
Protium opacum	Burseraceae	*Protium opacum*	Br	French Guiana	wet tropical	2	Levionnois *et al*. 2021
Prunus armeniaca	Rosaceae	*Prunus armeniaca*	Br	Cyprus	temperate	1	Crivellaro *et al*. 2012
Prunus avium	Rosaceae	*Prunus avium*	Br	Swiss	temperate	1	Crivellaro *et al*. 2012
Prunus domestica	Rosaceae	*Prunus domestica*	Br	Italy	temperate	1	Crivellaro *et al*. 2012
Pseudospondias microcarpa	Anacardiaceae	*Pseudospondias microcarpa*	Tr	Cameroon	wet tropical	1	Plavcová *et al*. 2024
Psydrax subcordata	Rubiaceae	*Psydrax subcordatus*	Tr	Cameroon	wet tropical	1	Plavcová *et al*. 2024
Pterocephalus multiflorus	Caprifoliaceae	*Pterocephalus multiflorus*	Tr, Br	Cyprus	mediterranean	2	Crivellaro *et al*. 2012
Punica granatum	Lythraceae	*Punica granatum*	Tr, Br	Cyprus	temperate	1	Crivellaro *et al*. 2012
Pycnanthus angolensis	Myristicaceae	*Pycnanthus angolensis*	Tr	Cameroon	wet tropical	1	Plavcová *et al*. 2024
Pyrus syriaca	Rosaceae	*Pyrus syriaca*	Tr, Br	Cyprus	temperate	1	Crivellaro *et al*. 2012
Qualea rosea	Vochysiaceae	*Qualea rosea*	Br	French Guiana	wet tropical	1	Levionnois *et al*. 2021
Quassia gabonensis	Simaroubaceae	*Quassia gabonensis*	Tr	Cameroon	wet tropical	1	Plavcová *et al*. 2024
Quercus coccifera	Fagaceae	*Quercus coccifera*	Br	Cyprus	mediterranean	1	Crivellaro *et al*. 2012
Quercus ilex	Fagaceae	*Quercus ilex*	Br	Italy	temperate	1	Crivellaro *et al*. 2012
Quercus petraea	Fagaceae	*Quercus petraea*	Tr	France	temperate	1	Adikurnia and Rathgeber 2025
Quercus pubescens	Fagaceae	*Quercus pubescens*	Tr	Slovenia	temperate	3	Gricar and Prislan 2022
Rauvolfia vomitoria	Apocynaceae	*Rauvolfia vomitoria*	Tr	Cameroon	seasonally dry tropical	2	Plavcová *et al*. 2024
Rhamnus alaternus	Rhamnaceae	*Rhamnus alaternus*	Tr, Br	Cyprus	mediterranean	1	Crivellaro *et al*. 2012
Rhus coriaria	Anacardiaceae	*Rhus coriaria*	Tr, Br	Cyprus	mediterranean	1	Crivellaro *et al*. 2012

Robinia pseudoacacia	Fabaceae	*Robinia pseudoacacia*	Br	Italy	temperate	1	Crivellaro *et al*. 2012
Rosa canina	Rosaceae	*Rosa canina*	Tr, Br	Cyprus	temperate	1	Crivellaro *et al*. 2012
Rosa x damascena	Rosaceae	*Rosa gallica*	Tr, Br	Cyprus	temperate	1	Crivellaro *et al*. 2012
Rosa chionistrae	Rosaceae	*Rosa chionistrae*	Tr, Br	Cyprus	temperate	1	Crivellaro *et al*. 2012
Salix alba	Salicaceae	*Salix alba*	Tr, Br	Cyprus	temperate	1	Crivellaro *et al*. 2012
Sambucus nigra	Viburnaceae	*Sambucus nigra*	Br	Swiss	temperate	1	Crivellaro *et al*. 2012
Sarcopoterium spinosum	Rosaceae	*Sarcopoterium spinosum*	Tr, Br	Cyprus	mediterranean	1	Crivellaro *et al*. 2012
Satureja thymbra	Lamiaceae	*Satureja thymbra*	Tr, Br	Cyprus	mediterranean	1	Crivellaro *et al*. 2012
Schumanniophyton magnificum	Rubiaceae	*Schumanniophyton magnificum*	Tr	Cameroon	wet tropical	1	Plavcová *et al*. 2024
Solanecio mannii	Asteraceae	*Solanecio mannii*	Tr	Cameroon	seasonally dry tropical	1	Plavcová *et al*. 2024
Spondia pinnata	Anacardiaceae	*Spondias pinnata*	Tr	China	wet tropical	2	XTBG, China
Staudtia kamerunensis	Myristicaceae	*Staudtia kamerunensis*	Tr	Cameroon	wet tropical	3	Plavcová *et al*. 2024
Sterculia tragacantha	Malvaceae	*Sterculia tragacantha*	Tr	Cameroon	wet tropical	2	Plavcová *et al*. 2024
Strombosia grandifolia	Olacaceae	*Strombosia grandifolia*	Tr	Cameroon	wet tropical	1	Plavcová *et al*. 2024
Strombosia postulata	Olacaceae	*Strombosia pustulata*	Tr	Cameroon	wet tropical	1	Plavcová *et al*. 2024
Styrax officinalis	Styracaceae	*Styrax officinalis*	Tr, Br	Cyprus	temperate	1	Crivellaro *et al*. 2012
Symphonia globulifera	Clusiaceae	*Symphonia globulifera*	Br	French Guiana	wet tropical	1	Levionnois *et al*. 2021
Syringa vulgaris	Oleaceae	*Syringa vulgaris*	Tr	Cyprus	temperate	1	Crivellaro *et al*. 2012
Syzygium staudtii	Myrtaceae	*Syzygium staudtii*	Tr	Cameroon	wet tropical	3	Plavcová *et al*. 2024
Tabernaemontana crassa	Apocynaceae	*Tabernaemontana crassa*	Tr	Cameroon	wet tropical	2	Plavcová *et al*. 2024
Tachigali melinonii	Fabaceae	*Tachigali melinonii*	Br	French Guiana	wet tropical	3	Levionnois *et al*. 2021
Tamarix tetragyna	Tamaricaceae	*Tamarix tetragyna*	Tr, Br	Cyprus	mediterranean	1	Crivellaro *et al*. 2012
Tamarix tetrandra	Tamaricaceae	*Tamarix tetrandra*	Tr	Cyprus	temperate	1	Crivellaro *et al*. 2012
Tapura africana	Dichapetalaceae	*Tapura africana*	Tr	Cameroon	wet tropical	1	Plavcová *et al*. 2024
Tetrapleura tetraptera	Fabaceae	*Tetrapleura tetraptera*	Tr	Cameroon	wet tropical	1	Plavcová *et al*. 2024
Teucrium cyprium	Lamiaceae	*Teucrium cyprium*	Tr	Cyprus	mediterranean	1	Crivellaro *et al*. 2012
Thottea barberii	Aristolochiaceae	*Thottea barberi*	Tr	India	wet tropical	1	Trueba *et al*. 2015
Thottea duchartreii	Aristolochiaceae	*Thottea duchartrei*	Tr	India	wet tropical	1	Trueba *et al*. 2015
Thottea ponmudiana	Aristolochiaceae	*Thottea ponmudiana*	Tr	India	wet tropical	1	Trueba *et al*. 2015
Thottea sivarajanii	Aristolochiaceae	*Thottea sivarajanii*	Tr	India	wet tropical	1	Trueba *et al*. 2015
Thymus capitatus	Lamiaceae	*Thymbra capitata*	Tr, Br	Cyprus	mediterranean	1	Crivellaro *et al*. 2012

Toona ciliata	Meliaceae	*Toona hexandra*	Tr	China	seasonally dry tropical	5	XTBG, China
Trichilia rubescens	Meliaceae	*Trichilia rubescens*	Tr	Cameroon	wet tropical	1	Plavcová *et al*. 2024
Sorindeia acuminata	Anacardiaceae	*Trichoscypha acuminata*	Tr	Cameroon	wet tropical	1	Plavcová *et al*. 2024
Turraeanthus africana	Meliaceae	*Turraeanthus africanus*	Tr	Cameroon	wet tropical	1	Plavcová *et al*. 2024
Ulmus pumila	Ulmaceae	*Ulmus pumila*	Tr	Mongolia	temperate	2	Dimitrova *et al*. 2024
Uvariodendron connivens,Uvariodendron connivers	Annonaceae	*Uvariodendron connivens*	Tr	Cameroon	wet tropical	2	Plavcová *et al*. 2024
Uvariodendron fuscum	Annonaceae	*Uvariodendron fuscum*	Tr	Cameroon	wet tropical	1	Plavcová *et al*. 2024
Uvariopsis dioica	Annonaceae	*Uvariopsis dioica*	Tr	Cameroon	wet tropical	1	Plavcová *et al*. 2024
Vepris afzelii	Rutaceae	*Vepris afzelii*	Tr	Cameroon	wet tropical	1	Plavcová *et al*. 2024
Viburnum tinus	Viburnaceae	*Viburnum tinus*	Tr, Br	Cyprus	mediterranean	2	Crivellaro *et al*. 2012
Vitex agnus-castus	Lamiaceae	*Vitex agnus-castus*	Tr, Br	Cyprus	mediterranean	1	Crivellaro *et al*. 2012
Vitex grandifolia	Lamiaceae	*Vitex grandifolia*	Tr	Cameroon	seasonally dry tropical	1	Plavcová *et al*. 2024
Zanthoxylum gilletii	Rutaceae	*Zanthoxylum gilletii*	Tr	Cameroon	wet tropical	3	Plavcová *et al*. 2024

Code for organs: Tr: Main stem samples; Br: Branch samples

Table S2 Anatomical slides preparing methods.

Method	Sample	Embedded	Sectioning method	Stain	Microscope
Crivellaro *et al*. 2012	Stem, microcores or disk	no	sliding microtome	Astra blue and safranin	transmission-light microscope
Plavcová *et al*. 2024	Microcores	no	15–20 µm sledge microtome	Astra blue and safranin	transmission-light microscope
Dimitrova *et al*. 2024	Microcores	in paraffin	9 µm semi-automatic rotary microtome	Astra blue and safranin	transmission-light microscope
XTBG, China	Microcores	in paraffin	8-10 µm rotary microtome	Astra blue and safranin	transmission-light microscope
Gricar and Prislan 2022	Microcores	in paraffin	8–12 µm rotary microtome	Astra blue and safranin	transmission-light microscope
Levionnois *et al.* 2021	Branch	in paraffin	8 µm rotary microtome	Alcian blue and safranin	transmission-light microscope
Adikurnia and Rathgeber 2025	Microcores	in paraffin	7 µm rotary microtome	Astra blue and safranin	transmission-light microscope
Trueba *et al.* 2015	Stem	no	blade cutting	Aqueous toluidine blue	transmission-light microscope

Table S3 Linear model regression between distance-to-tip and conduit diameters (D), density and fraction for combined dataset and samples from different laboratories (log (Traits) ~ log(distance)). Upper and lower 95% confidence intervals of model intercept and slope were shown in the table. When the separate model from the individual laboratory is significantly different from the combined data set model, the estimated parameters were in bold.

			Phloem Sieve Element Traits						Xylem Vessel Traits					
Organ	Trait	Method	Intercept	lower 95% CI	higher 95% CI	Slope	lower 95% CI	higher 95% CI	Intercept	lower 95% CI	higher 95% CI	Slope	lower 95% CI	higher 95% CI
Stem	hydraulic *D*	Combined dataset	2.43	2.37	2.49	0.26	0.23	0.29	3.59	3.51	3.68	0.39	0.35	0.43
		Crivellaro *et al.* 2012	2.38	2.3	2.46	0.18	0.1	0.26	3.55	3.45	3.66	0.27	0.17	0.37
		Plavcová *et al.* 2024	2.49	2.31	2.68	0.22	0.15	0.29	3.55	3.24	3.86	0.41	0.29	0.53
		XTBG, China	2.51	0.61	4.42	0.29	-0.32	0.89	4.44	2.62	6.25	0.2	-0.38	0.77
	upper quantile *D*	Combined dataset	2.49	2.43	2.55	0.26	0.23	0.29	3.63	3.54	3.72	0.39	0.35	0.44
		Crivellaro *et al.* 2012	2.44	2.36	2.52	0.18	0.1	0.26	3.6	3.49	3.71	0.25	0.15	0.36
		Plavcová *et al.* 2024	2.54	2.35	2.73	0.22	0.15	0.3	3.61	3.3	3.93	0.41	0.29	0.54
		XTBG, China	2.5	0.58	4.41	0.31	-0.3	0.92	4.53	2.6	6.45	0.19	-0.42	0.81
	median *D*	Combined dataset	2.38	2.32	2.44	0.26	0.23	0.29	3.41	3.32	3.5	0.4	0.36	0.44
		Crivellaro *et al.* 2012	2.33	2.25	2.4	0.18	0.1	0.25	3.36	3.25	3.46	**0.23**	**0.13**	**0.33**
		Plavcová *et al.* 2024	2.48	2.29	2.67	0.21	0.14	0.28	3.52	3.21	3.82	0.38	0.26	0.5
		XTBG, China	2.43	0.54	4.33	0.3	-0.3	0.91	4.46	2.6	6.33	0.15	-0.45	0.74
	Lower quantile D	Combined dataset l	2.28	2.22	2.34	0.26	0.23	0.29	3.14	3.05	3.23	0.38	0.34	0.42
		Crivellaro *et al.* 2012	2.22	2.14	2.29	0.17	0.09	0.24	3.07	2.97	3.17	**0.21**	**0.12**	**0.31**
		Plavcová *et al.* 2024	2.39	2.2	2.58	0.21	0.13	0.28	3.31	2.99	3.63	0.34	0.22	0.46
		XTBG, China	2.39	0.47	4.3	0.29	-0.32	0.89	4.61	1.99	7.23	-0.03	-0.86	0.81

	Density	Combined dataset	7.34	7.15	7.53	-0.39	-0.47	-0.31	5.22	4.86	5.58	-1	-1.16	-0.84
		Crivellaro *et al*. 2012	7.41	7.17	7.65	-0.31	-0.56	-0.06	5.35	5.02	5.68	-0.52	-0.86	-0.17
		Plavcová *et al*. 2024	7.09	6.49	7.7	-0.29	-0.52	-0.06	4.9	3.75	6.06	-0.96	-1.4	-0.52
		XTBG, China	7.33	1.17	13.49	-0.49	-2.38	1.4	2.48	-3.63	8.58	-0.29	-2.16	1.59
	fraction	Combined dataset	-1.7	-1.83	-1.57	0.06	0	0.12	-1.99	-2.22	-1.77	-0.17	-0.27	-0.08
		Crivellaro *et al*. 2012	-1.7	-1.84	-1.55	0.05	-0.1	0.21	-1.95	-2.17	-1.73	-0.01	-0.24	0.22
		Plavcová *et al*. 2024	-1.98	-2.39	-1.58	0.15	-0.01	0.3	-2.34	-3.12	-1.57	-0.09	-0.39	0.2
		XTBG, China	-2.03	-6.15	2.08	0.13	-1.13	1.39	-2.57	-7.43	2.29	0.01	-1.48	1.5
Branch	hydraulic *D*	Combined dataset	2.4	2.3	2.5	0.15	0.1	0.21	4.03	3.85	4.21	0.39	0.28	0.49
		Crivellaro *et al*. 2012	2.39	2.2	2.57	0.15	0.05	0.24	3.91	3.62	4.19	0.33	0.18	0.47
	upper quantile *D*	Combined dataset	2.46	2.36	2.56	0.15	0.1	0.21	4.05	3.86	4.23	0.37	0.26	0.47
		Crivellaro *et al.* 2012	2.45	2.26	2.63	0.15	0.05	0.24	3.86	3.56	4.15	0.27	0.12	0.43
	median *D*	Combined dataset	2.36	2.25	2.46	0.16	0.1	0.22	3.81	3.62	3.99	0.36	0.25	0.46
		Crivellaro *et al*. 2012	2.34	2.15	2.53	0.15	0.05	0.25	3.54	3.25	3.82	0.22	0.08	0.37
	Lower quantile D	Combined dataset	2.24	2.14	2.34	0.16	0.1	0.22	3.5	3.33	3.68	0.33	0.23	0.43
		Crivellaro *et al*. 2012	2.2	2.01	2.39	0.14	0.04	0.24	3.22	2.96	3.48	0.19	0.05	0.32

Table S4 PC loadings of 19 bioclimate factors.

		PC1	PC2	PC3
BIO1	Annual Mean Temperature	0.16	0.41	0.1
BIO2	Mean Diurnal Range	-0.26	0.03	0.28
BIO3	Isothermality	0.23	0.09	0.2
BIO4	Temperature Seasonality	-0.28	-0.14	0.01
BIO5	Max Temperature of Warmest Month	-0.2	0.33	0.13
BIO6	Min Temperature of Coldest Month	0.25	0.25	-0.08
BIO7	Temperature Annual Range (BIO5-BIO6)	-0.29	-0.08	0.12
BIO8	Mean Temperature of Wettest Quarter	0.15	0.22	0.5
BIO9	Mean Temperature of Driest Quarter	0.07	0.35	-0.43
BIO10	Mean Temperature of Warmest Quarter	-0.09	0.44	0.09
BIO11	Mean Temperature of Coldest Quarter	0.23	0.31	0.03
BIO12	Annual Precipitation	0.29	-0.09	-0.09
BIO13	Precipitation of Wettest Month	0.28	-0.07	-0.11
BIO14	Precipitation of Driest Month	0.24	-0.24	0.15
BIO15	Precipitation Seasonality (Coefficient of Variation)	-0.09	0.18	-0.35
BIO16	Precipitation of Wettest Quarter	0.28	-0.08	-0.11
BIO17	Precipitation of Driest Quarter	0.24	-0.22	0.15
BIO18	Precipitation of Warmest Quarter	0.25	0	0.37
BIO19	Precipitation of Coldest Quarter	0.27	-0.11	-0.22

Table S5 Partial regressions on response of trait to environment factors. Parameters estimated by GLS model on traits~PC1+PC2+PC3. Traits include diameters, density, and fraction. Akaike information criterion (AIC), Bayesian information criterion (BIC) and adjusted R-square of the model were shown. When P-value <0.05, the numbers were in bold.

Tissue	Trait	PC1	*P*-value	PC2	*P*-value	PC3	*P*-value	df	AIC	BIC	adjust R^2
sieve element	hydraulic diameter	-0.04	**0.04**	-0.04	0.06	0.1	**<0.001**	152	-1.39	13.86	0.14
	upper quantile diameter	-0.04	**0.03**	-0.04	**0.05**	0.1	**<0.001**	152	0.6	15.85	0.14
	median diameter	-0.04	0.06	-0.03	0.09	0.09	**<0.001**	152	-3.75	11.5	0.13
	lower quantile diameter	-0.03	0.14	-0.03	0.15	0.09	**<0.001**	152	-3.36	11.89	0.12
	density	0.05	0.39	-0.05	0.4	-0.12	**0.04**	80	135.29	147.45	0.02
	fraction	-0.05	0.23	-0.12	**<0.001**	0.01	0.89	80	60.79	72.94	0.12
vessel	hydraulic diameter	0	0.98	0.08	**<0.001**	0.06	**0.03**	152	117.13	132.38	0.07
	upper quantile diameter	0.06	**0.04**	0.15	**<0.001**	0.03	0.38	152	124.95	140.2	0.15
	median diameter	0.1	**<0.001**	0.16	**<0.001**	0	0.99	152	120.84	136.09	0.19
	lower quantile diameter	0.09	**<0.001**	0.15	**<0.001**	-0.01	0.75	152	128.42	143.66	0.17
	density	-0.34	**<0.001**	-0.4	**<0.001**	-0.23	**0.02**	80	222.63	234.79	0.3
	fraction	-0.2	**<0.001**	-0.18	**<0.001**	-0.01	0.92	80	153.23	165.38	0.18

References:

Adikurnia IK, Rathgeber CBK. 2025. Monitoring wood phenology using dendrometers: opportunities and pitfalls. *Quantitative Plant Biology* (accepted)

Crivellaro A. 2012. Wood, bark and pith structure in trees and shrubs of Cyprus: anatomical descriptions and ecological interpretation.

Dimitrova A, Balzano A, Tsedensodnom E, Byambadorj S-O, Nyam-Osor B, Scippa GS, Merela M, Chiatante D, Montagnoli A. 2024. The adaptability of Ulmus pumila and the sensitivity of Populus sibirica to semi-arid steppe is reflected in the stem and root vascular cambium and anatomical wood traits. *Frontiers in Plant Science* **15**: 1393245.

Gričar J, Prislan P. 2022. Seasonal changes in the width and structure of non-collapsed phloem affect the assessment of its potential conducting efficiency. *IAWA Journal*, **43**(3): 219-233.

Levionnois S, Salmon C, Alméras T, Clair B, Ziegler C, Coste S, Stahl C, González-Melo A, Heinz C, Heuret P. 2021. Anatomies, vascular architectures, and mechanics underlying the leaf size-stem size spectrum in 42 Neotropical tree species. *Journal of Experimental Botany* **72**: 7957–7969.

Plavcová L, Jandová V, Altman J, Liancourt P, Korznikov K, Doležal J. 2024. Variations in wood anatomy in Afrotropical trees with a particular emphasis on radial and axial parenchyma. *Annals of Botany* **134**: 151–162.

Trueba S, Rowe NP, Neinhuis C, Wanke S, Wagner ST, Isnard S. 2015. Stem Anatomy and the Evolution of Woodiness Piperales. *International Journal of Plant Sciences* **176**: 468–485.
